# FloorYOLO: an attention-enhanced YOLOv8n for floor plan object recognition

**DOI:** 10.3389/frai.2026.1911589

**Published:** 2026-09-15

**Authors:** S. Santhosh, M. S. Rashmika, A. Sasithradevi, Sabari Nathan

**Affiliations:** 1 School of Electronics and Computer Engineering, Vellore Institute of Technology, Chennai, Tamil Nadu, India; 2 Centre for Advanced Data Science, Vellore Institute of Technology, Chennai, Tamil Nadu, India; 3 Deloitte Tohmatsu DeepSquare Co., Ltd., Tokya City, Japan

**Keywords:** CVC-FP, deep learning, Efficient Channel Attention, floor plan detection, object detection, SESYD dataset, YOLOv8

## Abstract

**Introduction:**

The precise and automatic recognition and classification of structural elements like doors, walls, and windows, as well as categories of rooms in floor plan images, is crucial to the present-day application of real estate and architecture. Traditional approaches have the problems that the spatial relation is missing, the intra-class variance is high, dense packing is common, and clearly the boundary is hard to define, which has led to the development of efficient deep learning-based detection architectures.

**Methods:**

In this paper, a novel approach to automatic detection and localization of structural components on floor plans based on YOLOv8 with an Efficient Channel Attention (ECA) module is introduced. In this technique, YOLOv8 acts as the backbone model for detecting multiple classes, while the ECA block improves the representation of features by considering local interactions among channels without imposing significant extra computation. Moreover, the PAN neck enhances multilevel features with fine spatial information as well as semantic information in the image data, and the YOLOv8 head performs the detection task. The proposed system was tested using the SESYD dataset and the CVC-FP dataset, which include dense arrangements and scale variations in images.

**Results:**

Our model demonstrates improved discrimination between visually similar intraclass symbol variants that have proven difficult for both rule-based and deep learning approaches, with the proposed model achieving mAP@0.5:0.95 of 0.9794 and mAP@0.5 of 0.9950 on the 16-class SESYD benchmark, and mAP@0.5 of 0.8241 on CVC-FP against the baseline’s 0.8116.

**Discussion:**

The proposed FloorYOLO approach represents one of the first dual-dataset evaluations across synthetic and real-world floor plan benchmarks, demonstrating that lightweight channel attention can be effectively integrated into existing detection backbones to improve structural element recognition in floor plan imagery.

## 1 Introduction

The study of floor plans is considered an essential part of architectural designing, building information modeling, real estate management, and indoor navigation technologies (Xu et al., 2021). The ability to automatically identify architectural parts, such as windows, walls, doors, furniture, and rooms allows for efficient digitization and a deep understanding of building floor plans (Xu et al., 2021; Mewada et al., 2020). However, floor plans appear to be more challenging than natural images, because of their boundary-based nature, symbolic representation, and stylistic characteristics. Conventional rule-based and traditional image processing methods rely on handcrafted features, architectural assumptions and heuristic rules (Xu et al., 2021). Nevertheless, there are some drawbacks in the application of these techniques to complicated floor plans (Jung et al., 2023).

In recent times, deep learning has enabled object detection related techniques, such as Faster R-CNN.

and YOLO variants (Shehzadi et al., 2022; Mewada et al., 2020) to identify architectural elements in floor plans. Such methods yield good results and are suitable for real time use but only use physical feature-based characteristics. They do not pay attention to structural properties of floor plans, particularly room boundaries (Xu et al., 2025; Song and Yu, 2021). The spatial layout context strongly determines how the components of a plan are structured (Song and Yu, 2021). Typically, windows and doors are found on walls, furniture is in enclosed areas, and room semantics are described by bounded areas. These important boundary cues can make it much easier to confuse, localize and detect the object. This encourages the adoption of boundary-aware methods that guide feature learning toward structurally meaningful aspects (Xu et al., 2025; Song and Yu, 2021).

Several recent works have attempted to introduce this structural awareness into deep learning pipelines through attention-based mechanisms. Channel-wise feature recalibration, as introduced in Squeeze-and-Excitation Networks was shown to enable a network to concentrate on the most informative features while ignoring redundant ones, resulting in significant improvements for recognition-related tasks (Hu et al., 2018). Following this concept, MuraNet was extended to floor plan analysis with a relation-attention model that jointly segments and recognizes the symbols by considering the relatedness of structural components and objects located inside them (Huang et al., 2023). Other solutions have tackled the issue from a geometric perspective by transforming the image of the floor plan into a vectorized version that preserves the topological relationships, instead of relying on separate detections of walls, doors, and rooms (Song and Yu, 2021; Liu et al., 2017). Combined, these efforts suggest that lightweight attention mechanisms can be integrated into existing detection backbones to strike a balance between improving the model’s ability to recognize structurally similar symbols and avoiding the time-consuming geometric reconstruction pipelines.

To address these challenges, a novel method is proposed in this work, which integrates YOLOv8 and Efficient Channel Attention (ECA). The ECA block is added to the backbone architecture with the aim of enhancing its ability to extract features from the channel, while remaining cost-effective with minimal computation requirements. Through channel selection, the network better learns to discriminate between similar structures on the floor plan through channel selection. Multi-Level Feature Fusion is achieved using a neck component composed from PAN (Path Aggregation Network) architecture, which helps to ensure reliable detection results. We propose a framework which accepts an image of an unlabeled floor plan and outputs labeled structural elements.

## 2 Related works

With the recent development of vision based deep learning techniques, automatic floor plan analysis has become a more powerful means to improve the detection and localization of various structural components (Xu et al., 2021; Mewada et al., 2020). Advances in detection framework using the convolutional neural network (CNN) have been shown to learn intrinsic and hierarchical feature representations, achieving good performance (Shehzadi et al., 2022; Mewada et al., 2020). Recently, attention-based learning blocks have gained popularity for their ability of selectively learn salient spatial cues and contextual links in complex spaces. These methods are more applicable to images of the floor plan since they are less natural than images of the real world and have sparse texture and strong dependence on geometric structure and room boundaries (Jung et al., 2023; Xu et al., 2021; Song and Yu, 2021). The present section presents the main advances in the studies on floor plan analysis so far reported, strengths and weaknesses of the methods.

### 2.1 Traditional floor plan analysis methods

Rule-based floor plan analysis appears to be heavily influenced by certain rules that assess the floor plan. This is where rules on floor plan elements such as walls, rooms, and symbols are analyzed using assumptions made by people. Even though rule-based floor plan analysis is relatively effective for floor plans that are simple or standardized, it is considerably weak in terms of generalization (Lv et al., 2021; Ahmed et al., 2011). Ultimately, the aim of vectorization techniques is that they can be used for automatic conversion of a floor plan image represented as a raster image to a structural representation (Liu et al., 2017) like lines, polygons, and graphs. These structural representations are then converted to CAD format or BIM format. Vectorization techniques are very sensitive to noise and discontinuities and overlapping symbols (Wang et al., 2020; Sharma et al., 2017). Line detection techniques identify straight line segments related to the walls and boundaries using line detection algorithms or the Hough transform. They are the main part of the traditional floor plan parsing pipeline. However, broken lines, curves, and symbols lessen the line detection accuracy considerably. For this reason, line detection is considered an unsuccessful method (Lv et al., 2021; Feist et al., 2024). Erosion, Dilation, Opening and Closing are used to enhance the morphological components of a floor plan image. These processes have been applied to various applications to remove noise, join broken parts of walls, and to smoothen binary representations. Computational efficiency is assured for morphological approaches; however, they suffer from being very parameter-sensitive, but do not have semantic understanding of various architectural elements (Ahmed et al., 2011; Ravagli et al., 2019).

### 2.2 CNN based object detection methods

Convolutional Neural Network (CNN)—based object detection methods are fully automated but tend to deliver low-resolution results and blurred object boundaries. Some recent FCN-based extensions are concerned with these limitations: adaptive point-based rendering to obtain accurate boundary refinement (Pizarro et al., 2022) and attention-based design to learn pixel grouping and multi-scale contextual dependency (Cheng et al., 2020). U-Net is a fully convolutional encoder-decoder network with the capability of fusing deep semantic features with a fine-grained spatial detail via symmetric skip connections and achieving accurate pixel level semantic segmentation (Ziran and Marinai, 2018). The high boundary preserving ability has led to several developments, such as ensemble-based U-Net models for more robust models and higher segmentation accuracy in complex image scenes (Pan et al., 2017).

Aimed at solving the complex spatial layout and closely clustered architectural features, instance-aware inference of masks has been shown to work well for object-level detection and segmentation in floor plan images with the use of Mask R-CNN (Xu et al., 2025). For floor plan analysis the Mask R-CNN based frameworks utilize mask level supervision and intensive data augmentation methods to improve the robustness of the various numbers of annotations and different drawing styles, achieving accurate identification for structural components such as rooms, doors and symbols (Mishra et al., 2021). In general, CNN-based object detection techniques are very expensive in terms of computational cost and require extensive labeled training data to obtain high accuracy. They might end up performing differently if the input is low resolution, if the drawing is noisy, or if the floor plan style is different. Also, generalization across different datasets and drawing conventions is still a significant hurdle.

Objects including floor elements such as furniture, doors, windows and walls were detected in the SESYD dataset by using a deep neural network with a cascade mask R-CNN network (Shehzadi et al., 2022). YOLOv5 was used for numerous architectural floor plans, and the results were compared with existing methods like R-CNN; further challenges in working with small datasets were also investigated (Jung et al., 2023). A model was developed mainly for rural house floor plans, which featured diverse styles, and a dataset consisting of 800 annotated Chinese rural floor plans was created; the deep learning framework could predict room elements and textual information from the floor plans (Xu et al., 2021). A YOLOv5-based object detection approach was applied on the SESYD dataset for architectural floor plan analysis and room element detection; the dataset contains floor plan images with different types of noise and distortions, making object detection more challenging, and the detected outputs were utilized for applications such as converting 2D floor plans into structured digital or 3D models (Xu et al., 2025). A model took a novel approach to floor plan analysis by first vectorizing the images and then converting them into Region Adjacency Graphs; it was able to perform identification of room elements, like walls and windows, and spatial features like room corridors, and became a critical foundation for the development of further research (Song and Yu, 2021). A model was created to automatically extract meaningful information from floor plans and classifications, benefiting architects through the digitization of floor plan data for future use; the α-shape algorithm was used to calculate room areas, and a regression model was used to classify room types (Mewada et al., 2020). A deep learning-based framework for recognition and reconstruction of a residential floor plan was proposed. The structural elements and room components were detected using CNN and the floor plan was reconstructed to produce structured floor plan representations, where the advantages of deep learning for automated floor plan analysis are apparent (Lv et al., 2021). A floor plan automatic analysis system was also proposed to detect and label structural elements and rooms, and image processing techniques were used in conjunction with Optical Character Recognition (OCR) to extract and interpret textual labels in floor plans, which facilitated the identification of the various floor plan room types (Ahmed et al., 2011).

### 2.3 Modern object detection approaches

The recent advances that are being made in object detection techniques are mainly focusing on the contributions of deep convolutional neural networks, which are allowing the extraction of individual elements and learning. In object detection systems, two types of models are being used: single-stage and two-stage models. In two-stage models, the classification method is used, whereas in single-stage models, a single model is used. In recent times, object detection techniques are being improved, upgrading the accuracy of the system for varied areas of its usage, including the study of architectural floor plans (Shehzadi et al., 2022; Xu et al., 2021). YOLO is a single-stage object detection technique. In the YOLO object detection technique, object detection is carried out in a single-stage process. Also, unlike the region-based object detection technique, in the YOLO object detection technique, globally, over the overall image, object detection is carried out. This makes object detection in images highly efficient because globally, overall image object detection can be carried out, thereby making it possible to achieve object detection speed as well as employing the concept of context for object detection. Overall, object detection is achieved efficiently. Building on earlier single-stage detectors such as YOLOv3 (Redmon and Farhadi, 2018), in YOLO v4, balance has been achieved between object detection efficiency and object detection accuracy (Shehzadi et al., 2022; Jung et al., 2023).

Faster R-CNN uses a two-stage approach (Ren et al., 2015) and incorporates the RPN along with the CNN to achieve bounding boxes for the object of interest. The bounding boxes that can be obtained using the Faster R-CNN model can be further refined to achieve the output. Even though it achieved outstanding results and rightly detected objects of interest, the Faster R-CNN model was computationally complex compared to other single-stage detectors like YOLO. This model is considered a benchmark model to judge the efficiency of the network used for object detection (Xu et al., 2021).

Since 2023, object detection research has progressed rapidly, and floor plan analysis has started to keep pace rather than lag behind, contrary to what earlier studies in this field had anticipated. For complex architectural floor plans, Xu et al. (2021) proposed a YOLOv8-based architecture, ArchNetv2, in which a Convolutional Block Attention Module (CBAM) is incorporated to enhance feature learning for cluttered and visually similar objects such as stoves, bathtubs, and refrigerators. The closest published work to the attention placement investigated in this study applies attention within the backbone for floor plan analysis. Still, this line of work remains active and evolving, and typically relies on a heavier attention module within the backbone and neck, compared with embedding lightweight, parameter-free attention across both the backbone and neck in FloorYOLO. The study also highlighted that, even for auxiliary tasks such as label recognition, lightweight model selection remains important, OCR-based text spotting has been evaluated alongside object detection on architectural drawings in related work, comparing Faster R-CNN, YOLOv5, YOLOR, YOLOv7, and YOLOv8.

More broadly, the field of object detection continues to evolve in terms of what is considered lightweight. YOLOv10 completely eliminates the need for non-maximum suppression by introducing consistent dual label assignment, and the smaller YOLOv10-N and YOLOv10-S variants outperform their YOLOv6-3.0 counterparts while reducing the number of parameters by 51–61% and computational cost by 41–52%. This makes YOLOv10 one of the latest fully anchor-free, end-to-end YOLO variants to achieve an excellent balance between speed and accuracy. Although a speed difference still exists, RT-DETR has become highly competitive with advanced YOLO detectors. Specifically, the RT-DETR-R50 and RT-DETR-R101 variants achieve 53.1 and 54.3% AP on the COCO dataset at 108 and 74 FPS, respectively, on an NVIDIA T4 GPU. These developments indicate that the speed gap between transformer-based and CNN-based detectors has narrowed considerably. The remaining advantage of lightweight CNN detectors such as YOLOv8n therefore lies primarily in memory efficiency rather than inference speed.

### 2.4 Attention based object detection approaches

Attention mechanisms, originally introduced for sequence modeling through the Transformer architecture (Vaswani et al., 2017), have since been widely adapted into channel- and spatial-attention modules for vision tasks. YOLO-based detectors can detect objects with a single forward-pass inference, which makes them useful for efficient computational analyses of large-scale floor plans in real-time. Xu et al. (2021) and Mewada et al. (2020) noted that these detectors are particularly useful for identifying the recognition of the repetitive and standardized symbols found in architectural plans. YOLO also has recently introduced multi-scale feature extraction, which enhances the ability of detecting small and densely packed objects, like doors and fixtures in floor plans (Mewada et al., 2020). Jung et al. (2023) and Ravagli et al. (2019) show that targeted data augmentation methods significantly enhance the generalization ability of YOLO with respect to different floor plan drawing styles and inconsistencies in the annotation process.

Wang et al. (2020) addressed the issue of room classification in floor plans without textual information by proposing an improved C4.5 decision tree algorithm for room classification and by incorporating a detection method based on YOLOv3 to detect structural elements like doors and windows in floor plans and improving the classification results using the detected features. Sharma et al. (2017) proposed a deep learning-based framework, DANIEL (Deep Architecture for fiNdIng a likE Layouts), was proposed to automatically analyze and retrieve BFLs, which can extract low-level and high-level semantic features from BFL images for identifying and retrieving building layouts with similar architectural structures, with promising results compared with the state-of-the-art approaches. Feist et al. (2024) presented an up-to-date overview of automatic reconstruction of 3D models from 2D architectural drawings was given. Various existing approaches including deep learning, graph-based, and geometric processing techniques were analyzed for generating 3D building models from floor plans, highlighting challenges related to semantic extraction, geometric accuracy, and computational efficiency, as well as their importance in applications such as Building Information Modeling (BIM), interior design, and digital building visualization. Ravagli et al. (2019) proposed a floor plan text recognition, and classification method was proposed. It is focused on localizing, recognizing and categorizing textual information to retrieve the semantic building details of floor plans. Convolutional neural network (CNN) based methods were compared with traditional image processing methods, and several methods were integrated to enhance recognition results. The found text regions were segmented into four groups with respect to their functional purpose. Pizarro et al. (2022) comprehensively reviewed methods and approaches developed between 1995 and 2021, for automatic analysis of architectural floor plans to extract information from architectural floor plans are reviewed comprehensively. The rule-based method and the learning algorithm method were introduced in terms of datasets and scopes, providing support to future developers to increase their productivity and decrease the construction industry cost. The challenges of generalization and comparison due to the lack of standard metrics and public plan datasets were highlighted. Task combination was found to have potential applications for various applications. Ziran and Marinai (2018) adapted object detection in floor plan images, specifically identifying structural and semantic objects like furniture, doors, and windows to facilitate automated floor plan analysis and conversion to accessible digital forms. The paper focused on object detection architectures originally designed for natural images that were subsequently adapted for floor plan analysis and the datasets included floor plan images of various categories with respect to floor plan type for experimental evaluation. Pan et al. (2017) proposed a scheme of automatic detection and recognition of the floor plans was proposed based on structural elements like walls and windows. In the first step, the bearing walls were identified as non-bearing walls and in the second step, the window detection was done by applying contour-based analysis on non-bearing walls. The results were represented as structured floor plan representations which contained important architectural components for applications such as reconstruction and interior design.

Xu et al. (2021) noted that YOLO-based detectors can realize high inference efficiency but may not perform well under the condition of crowded layouts of the floor plan when compared to region-based detection frameworks. However, Mewada et al. (2020) pointed out that Faster R-CNN uses a region proposal network, which allows us to accurately localize the detected objects and is suitable for the detection of more complex architectural elements and the detection of spatially overlapping parts and Xu et al. (2021) added the two-stage detection process of Faster R-CNN is helpful for the accurate detection of overlapping and irregular symbols which is useful for performing deep analysis of the floor plan. But when compared to single-stage detectors such used by Jung et al. (2023) and Ravagli et al. (2019), Faster R-CNN is computationally costly and is not suitable for real-time analysis.

While Shehzadi et al. (2022), Jung et al. (2023), and Xu et al. (2025) have made significant advancements have been made in floor plan segmentation using CNN approaches and YOLO-type detectors, several important limitations persist. Current approaches to floor plan detection based on YOLO architectures ignore the necessity to re-calibrate each channel independently at both backbone and neck (PAN-FPN) stages. This becomes especially important given that floor plan images are texture-poor and contain intraclass symbols that are visually similar (four types of sinks and three types of tables in SESYD). Rezvanifar et al. (2020) noted that increasing information about boundary-related features while decreasing information about irrelevant features would lead to a lower number of misclassified objects, as was noted in earlier studies on floor plan symbol detection. Bochkovskiy et al. (2020) and Xu et al. (2024) have explored attention in terms of spatial and boundary-guided approaches while Jocher et al. (2023) and Huang et al. (2023) have investigated multi-task relation attention within segmentation pipelines; however, there are no consistent examples of using channel attention within both stages of feature extraction and multi-scale feature fusion processes. Hu et al.’s (2018) SENet-style channel attention relies on fully connected networks are too heavy-weight and therefore not applicable to lightweight versions of YOLO, such as YOLOv8n, that should be used for real-time purposes (for AR navigation or BIM integration) (Xu et al., 2021; Feist et al., 2024). The potential of FC-less, 1D adaptive convolution-based attention in this application scenario is yet to be explored (Shehzadi et al., 2022; Jung et al., 2023). In addition, most of the currently available solutions evaluate their performance on a single dataset, either SESYD or other custom datasets (Mewada et al., 2020; Wang et al., 2020), while cross-dataset generalization capabilities are investigated less (Xu et al., 2025; Pizarro et al., 2022).

To alleviate these gaps, our research makes the following contributions to the field of automatic floor plan object detection. To the best of our knowledge, no prior work has integrated the Efficient Channel Attention (ECA) module proposed by Wang et al. (2019) into YOLOv8n for architectural floor plan analysis existing YOLO-based detectors (Shehzadi et al., 2022; Jung et al., 2023) use standard backbones that treat all feature channels equally, which is a reasonable choice for natural images but poorly suited to the texture-sparse, boundary-driven characteristics of floor plan drawings. ECA is embedded at the output of every C2f block in both the backbone and the PAN-FPN neck, while the SPPF module is kept unmodified a placement confirmed by ablation experiments showing that ECA restricted to C2f layers alone yields the best precision–recall performance at both IoU@0.5 and IoU@0.5:0.95. To prevent thin boundary signals from being lost during channel recalibration, a residual skip connection termed ResECA is introduced, which preserves the original geometric edge detail that floor plan symbol structure fundamentally depends on (Xu et al., 2021; Song and Yu, 2021). Unlike SENet (Hu et al., 2018), which compresses channel information through fully connected bottleneck layers at significant parameter cost, the ECA mechanism uses a single adaptive 1D convolution with kernel size *k* < 9 and no dimensionality reduction, keeping the model within the computational budget required for real-time applications such as BIM integration and indoor AR navigation (Xu et al., 2021; Feist et al., 2024).

CoordAttention breaks apart the attention mechanism into two 1D pooling operations over height and width, capturing the position information, which is important in certain applications where the location of an object in the image is significant, such as natural-scene detection. Symbols on the floor plan, by comparison, are texture-sparse, boundary-defined and position independent: a door or window can be anywhere on the floor plan, and its identity is determined by the channel response of thin lines, rather than by spatial position. It does not seem to gain anything anymore for CoordAttention’ s added positional branch. In addition, CBAM adds another spatial attention branch, which our ablation study showed to be detrimental to the floor plan dataset (Section 4.3.3) because the branch weakens the already weak signal of thin lines in the channel. ECA’s single-branch, FC-free design is free from these problems, allowing for direct recalibration of channels without use of the SENet bottleneck or the additional spatial pathways of CBAM/CoordAttention.

## 3 Proposed methodology

The proposed architecture, FloorYOLO, is built on the YOLOv8n framework and uses Efficient Channel Attention (ECA) modules for all the C2f blocks in the backbone and PAN-FPN neck. The incorporation of ECA within the C2f modules allows the network to adaptively re-calibrate channel-wise feature responses, thereby emphasizing those features that convey more information and dampening less important ones. This attention mechanism makes the model more powerful to learn discriminative spatial and semantic information in multi-scale feature learning and fusion. Therefore, the network possesses greater ability to detect various types of floor plan symbols and structural components, which results in better detection performance and at the same time retaining the lightweight characteristics of the YOLOv8n architecture.

### 3.1 YOLOv8 model

YOLOv8 is a model capable of performing tasks such as object detection and segmentation in images. Its main developers of this model, Ultralytics, released it as an evolution of the YOLOv5 model with significant improvements in speed, accuracy, and versatility. One of the main properties of YOLOv8 is anchor-free detection. This indicates that the model does not rely on predefined anchor boxes for predicting object bounding boxes. Consequently, the model performs faster and more accurately, as it is not limited to specific box sizes. With such a feature, this technology can produce very precise results while allowing for rapid inference, and that is why it is a practical option for different types of object detection applications. As a single forward pass neural network, YOLOv8, locates objects by marking bounding boxes and class probabilities in one pass through the image. The model divides the image into an *n* × *n* grid and each cell is a contributor to the prediction of bounding boxes and class probabilities. After completing the predictions, the model uses Non-Maximum Suppression (NMS) to remove any overlap between bounding boxes so that only the most confident predictions remain. In this research work, once an input image is processed by the model, features are extracted using convolution and pool layers, guided by the annotated dataset.

YOLOv8 is composed of three main stages. The backbone is built on the modified CSPDarknet with C2f modules and extracts hierarchical feature representations from the input image with growing receptive fields. The neck is a Path Aggregation Network (PANet) with Feature Pyramid Network (FPN)-style connections (Lin et al., 2017), which aggregates multi-scale features from the backbone to facilitate accurate detection of small and large objects. We employ a decoupled head that predicts bounding box coordinates and class probabilities separately on three different output scales. Figure 1 illustrates this overall pipeline, tracing the input image through the stem and backbone C2f blocks, the SPPF module, the PAN-FPN neck, and finally the decoupled detection head. The FPN consists of several convolutional layers that extract features at different resolutions, improving the model’s ability to recognize objects of different sizes. YOLOv8 uses the SiLU (Sigmoid Linear Unit) activation function as the main activation function throughout the model, also known as Swish, defined as:


f(x)=x·σ(x),whereσ(x)=1/(1+e^(−x)) (1)

**Figure 1:**
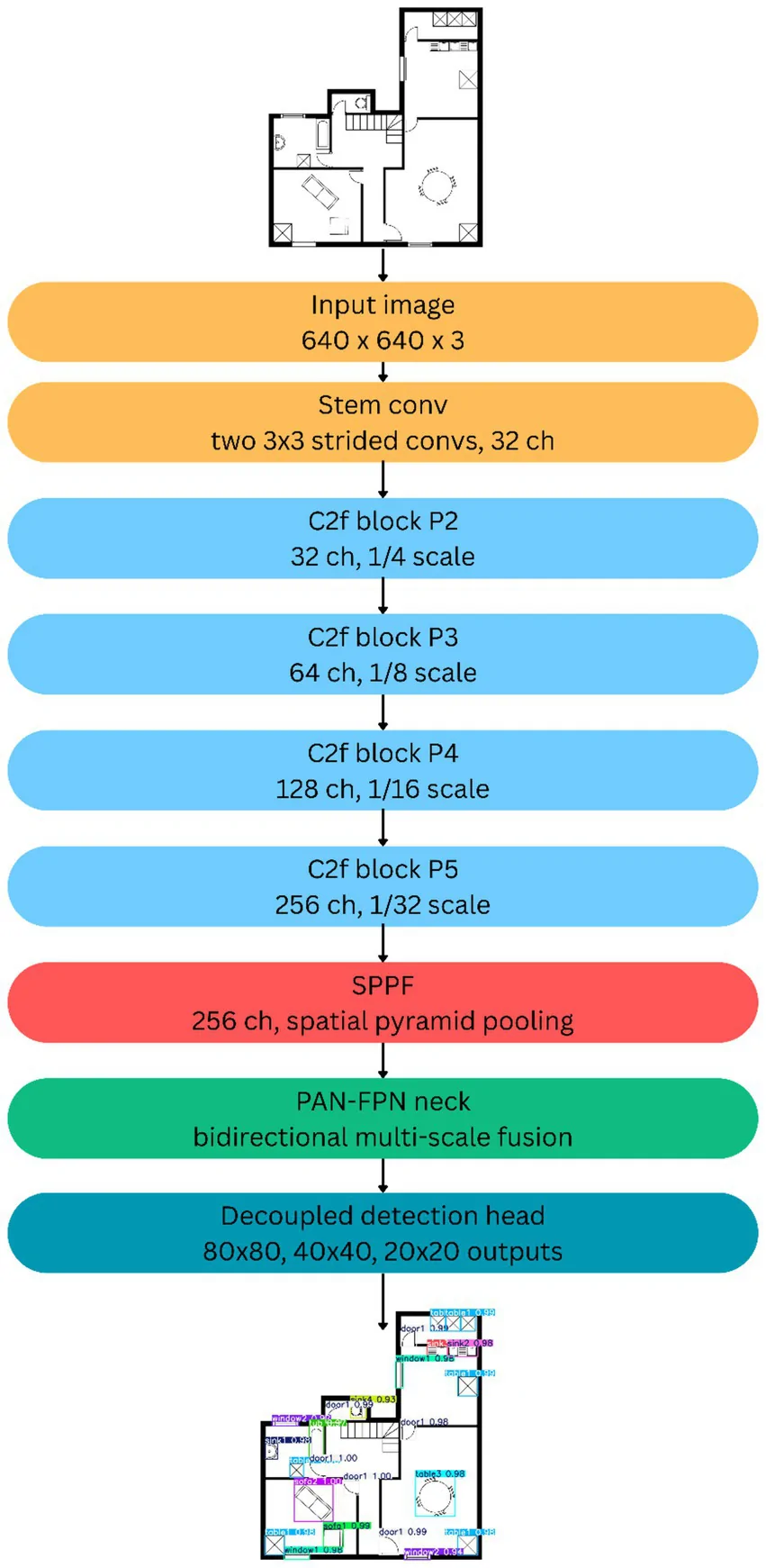
YOLOv8n architecture overview showing backbone, neck (PAN-FPN), and detection head.

This smooth, non-monotonic behavior, as given in Equation 1, is why SiLU is favored over alternatives like Leaky ReLU, helping to ease the vanishing gradient problem and allow for steadier training.

### 3.2 Attention mechanism

The Efficient Channel Attention (ECA) module enables us to refine the features based on the channel with low runtime costs while maintaining the lightness of the model. Attention of this kind helps the model to differentiate various architectural elements, such as doors, walls, and windows. The ECA mechanism uses global average pooling to transform an intermediate feature map input which measures 𝐶 × 𝐻 × 𝑊 dimensions into a channel descriptor. This descriptor is processed by a fast 1D convolution to extract local cross channel interaction and produces channel attention weights using a sigmoid activation function. The weights allow the original feature map to be recalibrated by element-wise multiplication to yield a refined representation of the feature map. The output of the ResECA residual structure is generated by merging the original input feature map with the refined feature map. This full sequence, pooling, adaptive 1D convolution, sigmoid weighting, and residual recalibration, is laid out in Figure 2. Adaptive kernel sizes are used in the 1D convolution, to achieve the effective channel interaction between neighboring channels with minimal operation cost for feature representation. ECA is the perfect solution for real-time object detection applications, based on the SPPF and C2f modules.

**Figure 2:**
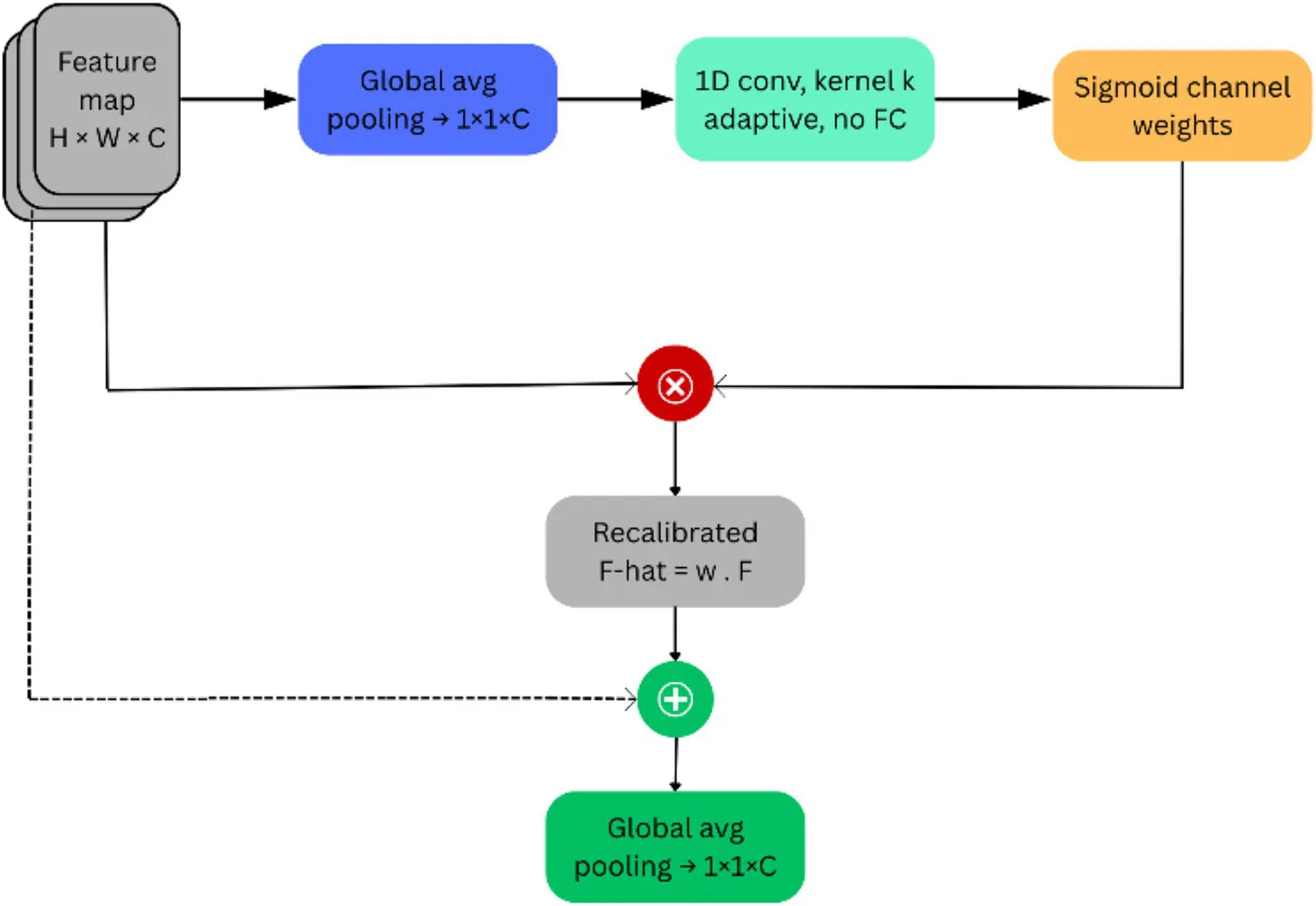
Architecture of the Efficient Channel Attention (ECA) module with a residual skip connection (ResECA).

#### 3.2.1 Global average pooling

The spatial compression of the input feature map *F* ∈ *ℝ*^(*C* × *H* × *W*) as given in Equation 2:


$$z_{c}=(\frac{1}{H\times W})\sum_{i=1}^{H}\sum_{j=1}^{W}F_{c}\sum F_{c(i,j)},\text{for}\;c=1,2,\ldots ,C$$ (2)

where *z_c_* is the channel descriptor for channel *c*. Here, the input tensor *F* ∈ ℝ^(*C* × *H* × *W*) will be mapped to output descriptor *z* ∈ ℝ^(*C* × 1 × 1), which means that the spatial dimensions *H* and *W* are collapsed, but the channel dimension *C* is preserved.

#### 3.2.2 1D convolution with adaptive kernel

The weights for the channels are calculated from the descriptor *z* ∈ ℝ^*C* as given in Equation 3, is:


$$\omega =\sigma (\text{Conv}1D_{k}(z))$$ (3)

where *σ* is the sigmoid function, in this case, *z* ∈ ℝ^*C* is transformed into ℝ^(1 × *C*) in order to work as a one-dimensional input signal for the 1D convolution. In this 1D convolution, a single shared filter with width *k* is used, resulting in attention weights *ω* ∈ ℝ^(*C* × 1 × 1) where each *ω*_*c* ∈ (0,1). Due to the usage of just *k* parameters for the filter and also the fact that it does not change the dimensionality of channels, the computational cost becomes *O*(*k*) instead of *O*(*C*^2^). The value of *k* is the adaptive kernel size which is defined in Equation 4:


$$k=\psi (C)={|\frac{\log2(C)}{\gamma }+\frac{b}{\gamma }|}_{\mathrm{odd}}$$ (4)

where *γ* and *b* are hyperparameters (usually *γ* = 2, *b* = 1), and |·| odd rounds to the nearest odd number.

#### 3.2.3 Channel recalibration (element-wise multiplication)

The original feature map is rescaled using the attention weights, as given in Equation 5:


$$\hat{F_{c}}=\omega_{c}\cdot F_{c}$$ (5)

where *F̂* is the recalibrated feature map.

The weight vector *ω* ∈ ℝ^(*C* × 1 × 1) is broadcast along spatial axes to multiply with the input element wise, resulting in *Ḟ* = *ω* ⊙ *F* ∈ ℝ^(*C* × *H* × *W*), which is the same as the shape of the input tensor *F*. This example demonstrates that channel recalibration reweights feature information without altering the tensor shape.

#### 3.2.4 ResECA skip connection

The final product is a re-calibrated map along with the original input, as given in Equation 6:


$$F_{\text{out}}=\hat{F}+F=\omega \cdot F+F=(\omega +1)\cdot F\;$$ (6)

Output *F*_out ∈ *ℝ*^(*C* × *H* × *W*) perfectly matches the input tensor from the dimension standpoint, allowing one to include ResECA into the architecture right after any C2f layer in the backbone and neck parts of the network without any extra operations such as reshaping, projection, etc., consistent with the residual learning principle (He et al., 2016).

### 3.3 Architecture of FloorYOLO model

The architecture adds a single ECA module to the output of every C2f block along both the backbone and the PAN-FPN neck of YOLOv8n. The full pipeline, from the input floor plan through the backbone, neck, and detection head, is shown in Figure 3. This placement strategy diverges from the more typical approach of limiting attention to the deepest backbone stages. That method generally works well for natural image datasets, where high-level features dominate the detection tasks. Though, floor plan imagery presents a different challenge. Here, crucial details like the arc of a door swing, the notch of a window recess, and the slight difference in sink shapes are geometric and fine-grained, best captured in the shallower, higher-resolution feature maps. Initial tests on the SESYD validation set supported this finding. Limiting ECA to P4 and below led to consistent drops in recall for door and window classes. Expanding its use to all C2f layers recovered those missed detections without sacrificing precision elsewhere. This informed the final design choice of placing ECA uniformly across the network (see Figure 4).

**Figure 3:**
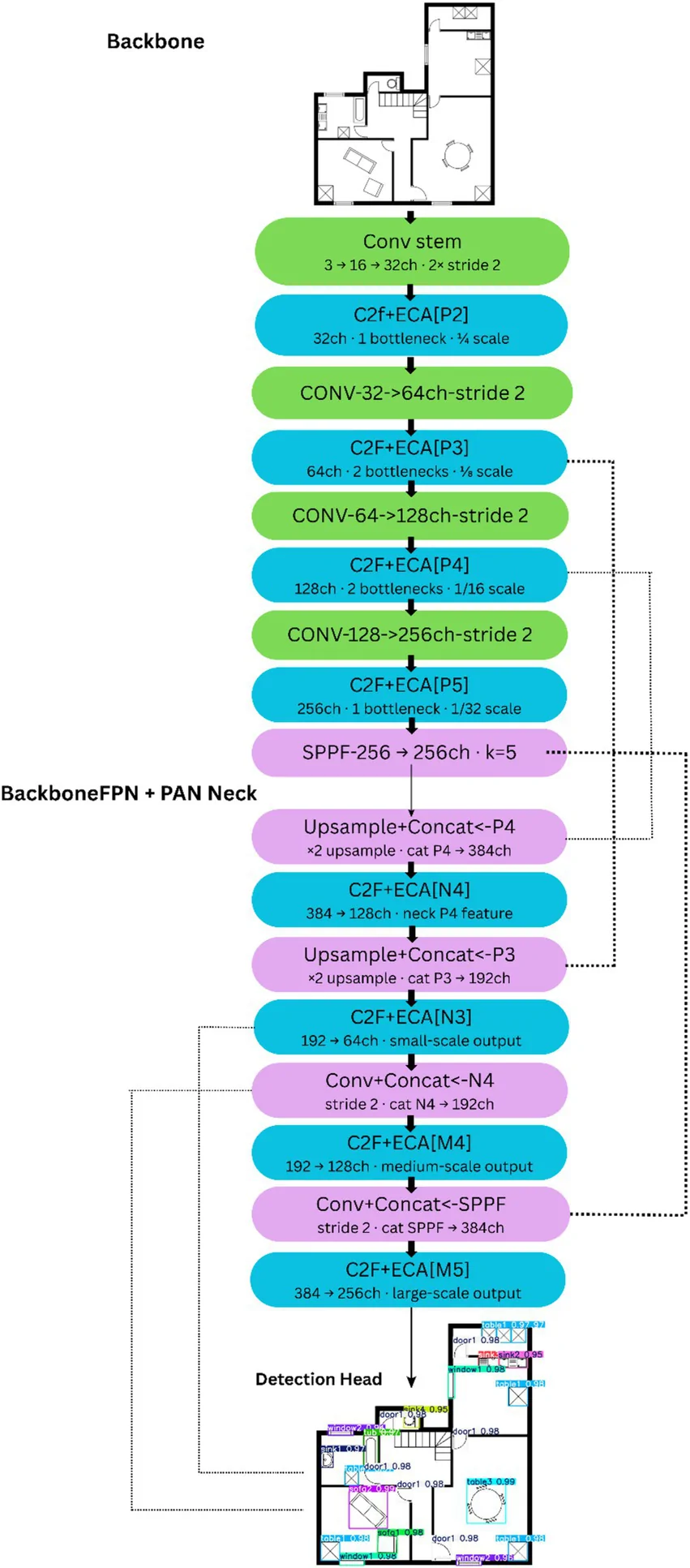
Detailed architecture of FloorYOLO.

**Figure 4:**
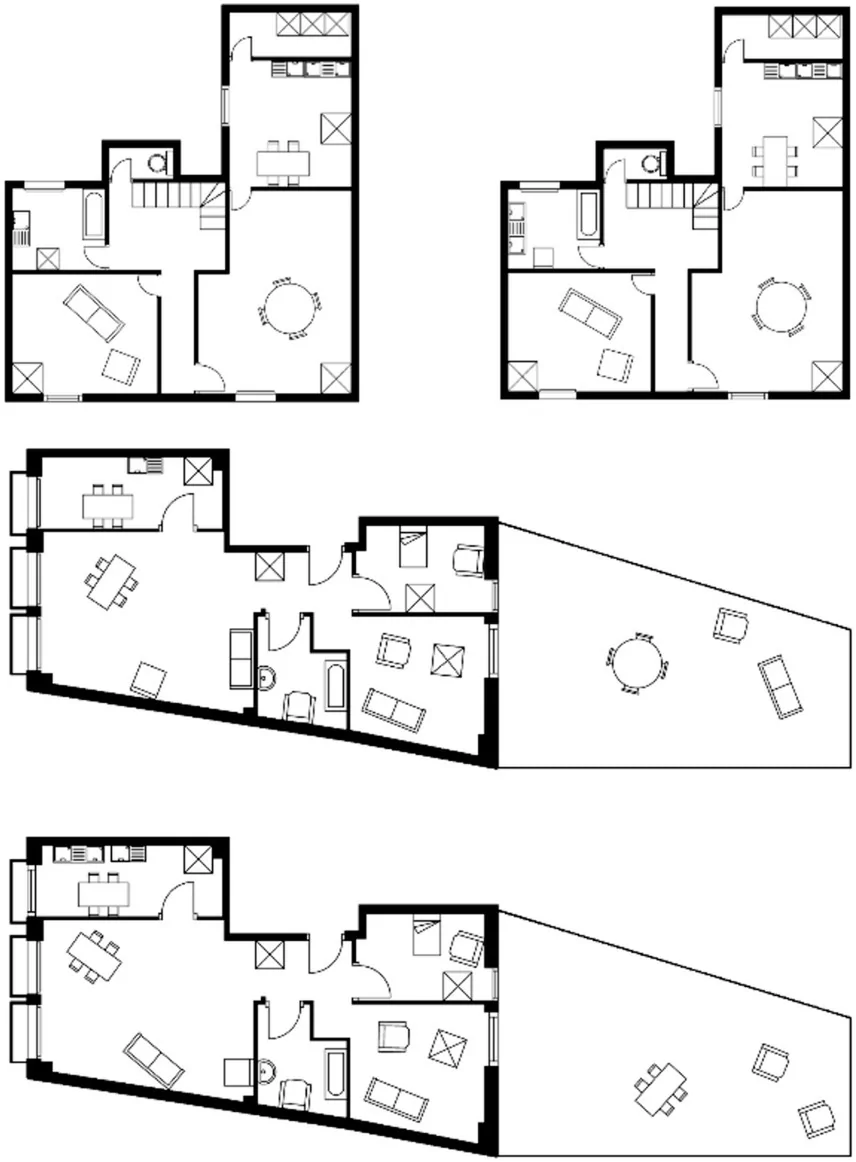
Sample SESYD floor plan images.

The backbone starts with a two-stage convolutional stem that processes the 640 × 640 × 3 input and generates a 32-channel feature map at half the original resolution via two consecutive 3 × 3 strided convolutions. This is followed by four C2f-ECA stages, each separated by strided down sampling convolutions that gradually double the channel count: P2 has 32 channels at a ¼ scale (1 bottleneck), P3 has 64 channels at an ⅛ scale (2 bottlenecks), P4 has 128 channels at a ^1^/₁₆ scale (2 bottlenecks), and a deeper stage has 256 channels at a ^1^/₃₂ scale (1 bottleneck). Within each block, the C2f module first applies its split-concat bottleneck operations to extract local spatial features. Then, the ECA module recalibrates the channel responses before the output moves downstream. Since ECA uses a single 1D convolution with an adaptive kernel of no more than 9 parameters and does not reduce dimensionality, this recalibration step adds almost no extra overhead at each stage compared to the following convolutions.

The ResECA skip connection, explained in Section 3.2, is critical in the shallower stages. Early feature maps carry thin-stroke boundary signals, wall lines, hatch patterns, and symbol outlines that are geometrically precise but have low energy. Aggressive channel gating without a residual bypass risks weakening these low-energy boundary channels in favor of stronger but less distinctive interior activations. The identity branch of the skip connection prevents this: even if a boundary-sensitive channel receives low attention, its contribution is still added to the output. This maintains the geometric details that differentiate, for example, door1 from door2 at the bounding-box level.

The backbone ends with an unmodified SPPF block at 256 channels, which uses cascaded 5 × 5 max pooling at three scales to expand the effective receptive field before the features enter the neck. Testing ECA after the SPPF was conducted and rejected. The block already processes globally pooled context, and channel recalibration at that point made no noticeable difference in either mAP@0.5 or mAP@0.5:0.95 across experiments. Leaving SPPF unchanged also keeps a clear boundary between the backbone’s role in contextual aggregation and the neck’s role in bridging scales.

The neck follows a bidirectional PAN-FPN structure, featuring C2f-ECA blocks at each fusion point. In the top-down path, the SPPF output is up sampled by a factor of two and concatenated with the 128-channel P4 feature map, creating a 384-channel tensor to be processed by C2f-ECA block N4 into a 128-channel representation. The same operation applies one scale lower: N4 is upsampled and concatenated with the 64-channel P3 map (192 channels total), with C2f-ECA block N3 reducing this to the 64-channel small-scale output. Placing ECA at these fusion points is intentional; cross-scale concatenation merges feature maps with very different semantic depths. Not all resulting channels provide equally useful information for subsequent tasks. Without recalibration, channels from the shallower map that convey generic low-level texture, which floor plans generally lack, can dilute the semantically richer channels from the deep backbone. The C2f-ECA blocks at each fusion step counter this dilution effectively. The bottom-up path reflects this logic in reverse. A strided convolution on N3 connects with N4 (192 channels), which C2f-ECA block M4 refines into the 128-channel medium-scale output. A further stride on M4 combines with the SPPF output (384 channels), from which C2f-ECA block M5 generates the final 256-channel large-scale feature map. Applying attention in both directions of the pyramid, rather than just at the top or the bottom, guarantees that channel selectivity is enforced both when passing semantic context downward and when re-aggregating spatial detail upward. This approach creates cleaner representations for each scale and reduces the feature ambiguity that arises when the same network needs to differentiate between objects that are spatially as distinct as a bathtub and a window tag within the same image.

The three output feature maps, sized 80 × 80, 40 × 40, and 20 × 20, go to the decoupled detection head, which has separate convolutional branches for classification and bounding-box regression. The regression branch employs Distribution Focal Loss (DFL) to model bounding-box edges as discrete probability distributions rather than point offsets. This improves localization precision for symbols with compact, well-defined extents. Decoupling the two prediction tasks keeps gradient conflicts between classification and localization from happening during training. This is an important consideration when both tasks are challenging at the same time. Differentiating sink3 from sink4 is a fine-grained classification task, while tightly localizing a small armchair in a heavily annotated floor plan presents a precision-localization challenge. Managing them through independent branches, supported by consistently channel-recalibrated features from the neck, allows the model to optimize each objective without compromising the other.

## 4 Experiment and results

### 4.1 Experimental setup

The experiments for this study were conducted on the Google Colab cloud computing service, which enables GPU processing. The implementation framework is using a PyTorch backend and the YOLOv8n model from the Ultralytics YOLOv8 framework has been used in Python 3 programming language. All experiments were conducted using Ultralytics YOLOv8 (v8.4.53), PyTorch 2.10.0 (CUDA 12.8 build), and Python 3.12.13 run on the Tesla T4 GPU provided via Google Colab. All the models have been trained using the AdamW optimizer (automatically chosen by Ultralytics optimizer resolution depending on the model and dataset used), using a fixed seed of 0 for all experiments and deterministic training. The reason for selecting the YOLOv8n model is its lightweight structure and efficiency in performing the required tasks. In this study, the SESYD dataset was used for training, which has 700 images in the training set and 150 images in the validation and test sets. Additionally, the CVC-FP dataset was also used, which contains 98 images in the training set and 25 images in the validation set. These were defined using a YAML file. An appropriate set of hyperparameters such as learning rate, batch size, optimization algorithm selection, and epoch selection for convergence was chosen to guarantee stability and enhance the model’s object detection accuracy. Training was carried out in 30 epochs using images with a resolution of 640 × 640 pixels. The use of pre-trained weights assisted in improving the convergence and accuracy of the model. The training hyperparameters used in this study are summarized in Table 1.

**Table 1:** Training parameters.

Parameter	Configuration
Learning rate	0.001
weight decay	0.0005
Batch size	16
optimizer	AdamW
Image size	640 × 640
Epochs	30

All baseline and ablation models (YOLOv8n, augmented CBAM-YOLOv8n, dual block ECA, and YOLOv10n/v11n/v12n/v26n) in Tables 2, 3, 4 were re-trained based on the same process explained in Table 1 (30 epochs, 640 × 640 image size, AdamW optimizer, same weight initialization procedure), using the exact same train/validation/test set of the SESYD data set, as explained in section 4.1. The only exception is Tables 5, 6, where results are from other published papers.

**Table 2:** Ablation study results on SESYD dataset.

Model	Split	mAP@0.5	mAP@0.5:0.95	Precision	Recall
Baseline (YOLOv8n)	Test	0.995	0.974	0.9973	0.9989
Yolov8n with ECA in all C2F and SPPF layers	Test	0.995	0.9754	0.9926	0.9989
Yolov8n with Vanilla ECA in backbone (no residual)	Test	0.995	0.976	0.996	0.998
Yolov8n with ECA in Neck only	Test	0.995	0.972	0.996	0.999
Yolov8n with ECA in Backbone only	Test	0.995	0.976	0.996	0.998
Yolov8n with CBAM	Test	0.9869	0.8724	0.9633	0.9582
FloorYOLO	Test	0.995	0.9794	0.9984	0.9994

**Table 3:** Comparison with existing solutions for SESYD floor plan detection methods.

Title	Author (Year)	Model	Dataset	mAP@0.5
Object detection in floor plan images	Ziran and Marinai (2018)	Faster R-CNN variants	SESYD (d1/d2)	0.31–0.39
Symbol spotting on digital architectural floor plans using a deep learning-based framework	Rezvanifar et al. (2020)	YOLOv2 + ResNet-50 (tiling)	SESYD	~ 0.981
Towards robust object detection in floor plan images: a data augmentation approach	Mishra et al. (2021)	Cascade Mask R-CNN + deformable conv	SESYD + SFPI	0.982
Mask-aware semi-supervised object detection in floor plans	Shehzadi et al. (2022)	Mask R-CNN semi-supervised (student-teacher)	SESYD	~ 0.995

**Table 4:** Comparison of YOLO variants on SESYD dataset.

Model name	mAP50-95	mAP50	Precision	Recall
Yolo v10n	0.954	0.9735	0.9268	0.9047
Yolo v11n	0.9749	0.9949	0.9967	0.9968
Yolo v12n	0.9736	0.9950	0.9944	0.9970
Yolo v26n	0.9204	0.9950	0.8991	0.8816
FloorYOLO model	0.9794	0.9950	0.9984	0.9994

**Table 5:** Performance on hard subsets of the SESYD test set.

Model	Subset	Images	Precision	Recall	mAP@0.5	mAP@0.5:0.95
Baseline (YOLOv8n)	Small (<32 × 32px)	145	0.9973	0.9988	0.995	0.9742
Baseline (YOLOv8n)	Overlap	134	0.9968	0.9997	0.995	0.9777
Baseline (YOLOv8n)	Low contrast	30	0.9939	1	0.995	0.9867
FloorYOLO (ECA)	Small (<32 × 32px)	145	0.9979	0.9995	0.995	0.9777
FloorYOLO (ECA)	Overlap	134	0.9975	1	0.995	0.981
FloorYOLO (ECA)	Low contrast	30	0.9949	0.9984	0.995	0.9866

**Table 6:** Computational efficiency and accuracy comparison on the SESYD dataset.

Model name	Params (M)	GFLOPs	GPU FPS	CPU FPS	mAP@0.5:0.95
Baseline (YOLOv8n)	3.014	8.208	97.79 ± 10.21	6.60 ± 0.65	0.974
FloorYOLO (ECA)	3.014	8.208	96.66 ± 14.22	6.26 ± 0.53	0.9794
YOLOv10n	2.713	8.423	65.92 ± 5.73	5.61 ± 0.64	0.9551
YOLOv11n	2.593	6.454	67.76 ± 8.39	6.84 ± 0.79	0.9752
RT-DETR-L	32.839	109.958	21.51 ± 0.53	0.59 ± 0.01	0.9911
MobileNet-SSD	2.414	—	53.63 ± 4.17	13.92 ± 1.28	0.7723
NanoDet-m	0.95	0.72	54.69	35.95	0.451

### 4.2 Evaluation metrics

#### 4.2.1 Mean Average Precision

Mean Average Precision (mAP) is the standard metric for evaluating object detection performance, computed by averaging the precision across all classes in the dataset. In Equation 7, APs denotes the Average Precision for class *S*, and *S* is the number of classes in the data set. The area under the curve obtained by plotting precision against recall on a graph over a range of confidence thresholds of the detector is calculated for each class. The higher the AP, the more confident a class is and the lower its error, over a range of operating points, not at a single fixed AP. The floor plan datasets SESYD and CVC-FP also include a mix of frequent (wall, door) and rare (speculative furniture types) symbols. As such, mAP is reported over these datasets to avoid being dominated by performance on the high frequency classes and avoid masking poor performance on the low frequency classes.


$$\mathrm{mAP}=\frac{1}{S}\sum_{S=1}^{S}{\mathrm{AP}}_{S}\;\;\;\;$$ (7)

#### 4.2.2 Precision

The ratio of true positives to the total of false positives and true positives is known as precision as expressed in Equation 8 (Wang et al., 2020).

In this case TP (true positive) means a symbol that is correctly detected, and FP (false positive) means a symbol that is incorrectly or spuriously detected. A false positive in the context of the detection of floor plan symbols is usually the case when the model classifies a symbol type that does not exist in the plan or when the model classifies a type of symbol into a different class, e.g., recognizing a table as a sink, which is more likely to happen because of the intraclass visual similarity of symbols discussed in Section 2.4. Precision, in turn, is a direct measure of the true positive detection rate, so that low precision would result in a high percentage of detections needing to be manually checked downstream, such as in generating BIM or room labelling, thereby nullifying the benefit of automation.


$$\text{Precision}=\frac{\mathrm{TP}}{\mathrm{TP}+\mathrm{FP}}$$ (8)

#### 4.2.3 Recall

Recall is the ratio of true positives to the sum of true positives and false negatives (Wang et al., 2020).

FN (false negatives) in Equation 9 represent symbols that exist in the ground truth but are not detected. The false negative is the case where a symbol that should be included in the floor plan is not detected at all, which is particularly consequential for structural symbols like doors and windows; the downstream tasks like AR navigation or room segmentation can be affected by a missed door. Recall is not a measure of accuracy, but rather of completeness, thus, there is a tradeoff: a detector with a high recall rate will tend to have a low precision rate, and a detector with a high precision rate will tend to have a low recall rate. The trade off the model makes at a given level of confidence is provided by reporting both Equations 8, 9 together, not separately.


$$\text{Recall}=\frac{\mathrm{TP}}{\mathrm{TP}+\mathrm{FN}}$$ (9)

### 4.3 Performance evaluation of FloorYOLO model on the SESYD dataset

#### 4.3.1 SESYD dataset

The SESYD dataset consists of synthetic floor plan images, resembling architectural drawings. It contains 1,000 images of floor plans with complex layouts, each labelled with a symbol, with 16 classes of symbols: armchair, bed, door1, door2, sink1, sink2, sink3, sink4, sofa1, sofa2, Tables 1, 2, 5, tub, window1, window2. The majority of symbol classes are related to the furnishings and/or equipment of residential/domestic buildings, and not to the full spectrum of symbols that may be found in other building types. Further, SESYD has 1,000 images, but it is synthetic, so there is less structural diversity, compared to what is commonly found in architectural drawings that are actually scanned. SESYD is therefore treated as a controlled benchmark for symbol-detection accuracy rather than a representative sample of real-world architectural diversity. This limitation is partially addressed through the use of the CVC-FP dataset (Section 4.4), which comprises scanned real-world floor plans with greater structural and graphical variability.

#### 4.3.2 Evaluation

The floor-plan dataset is used with the ECA based network and YOLOv8n. In our experiment 70% of the floor-plan images are used to train the model, 15% to test and 15% to validate the model. The average precision for each class is shown in Figure 5. Most of the classes have very high mean precision values near to 1, and the rest of the classes, except window1 and window2, have mean precision values above 0.95. The overall performance of the model achieved mAP@0.5 of 0.9950 and mAP@0.5:0.95 of 0.9794, with Precision of 0.9984 and Recall of 0.9994, consistent with the F1-confidence curve in Figure 6, which holds a combined F1 score of 1.00 across most of the confidence range before dropping off near a confidence of 0.594. Representative qualitative outputs on the SESYD test images are shown in Figure 7. The unmodified YOLOv8n baseline model was trained under the same protocol as described in Table 1, and on SESYD an mAP@0.5 of 0.9950, mAP@0.5:0.95 of 0.974, Precision of 0.9973, and Recall of 0.9989 (Table 2). The improvement of FloorYOLO over this baseline is in mAP@0.5:0.95 (0.9794 vs. 0.974) and precision (0.9984 vs. 0.9973) but not mAP@0.5, as both models have already reached near-saturated results on this controlled synthetic dataset, which is not a uniform baseline training problem but rather due to the limited structural diversity of the dataset (Section 4.3.1).

**Figure 5:**
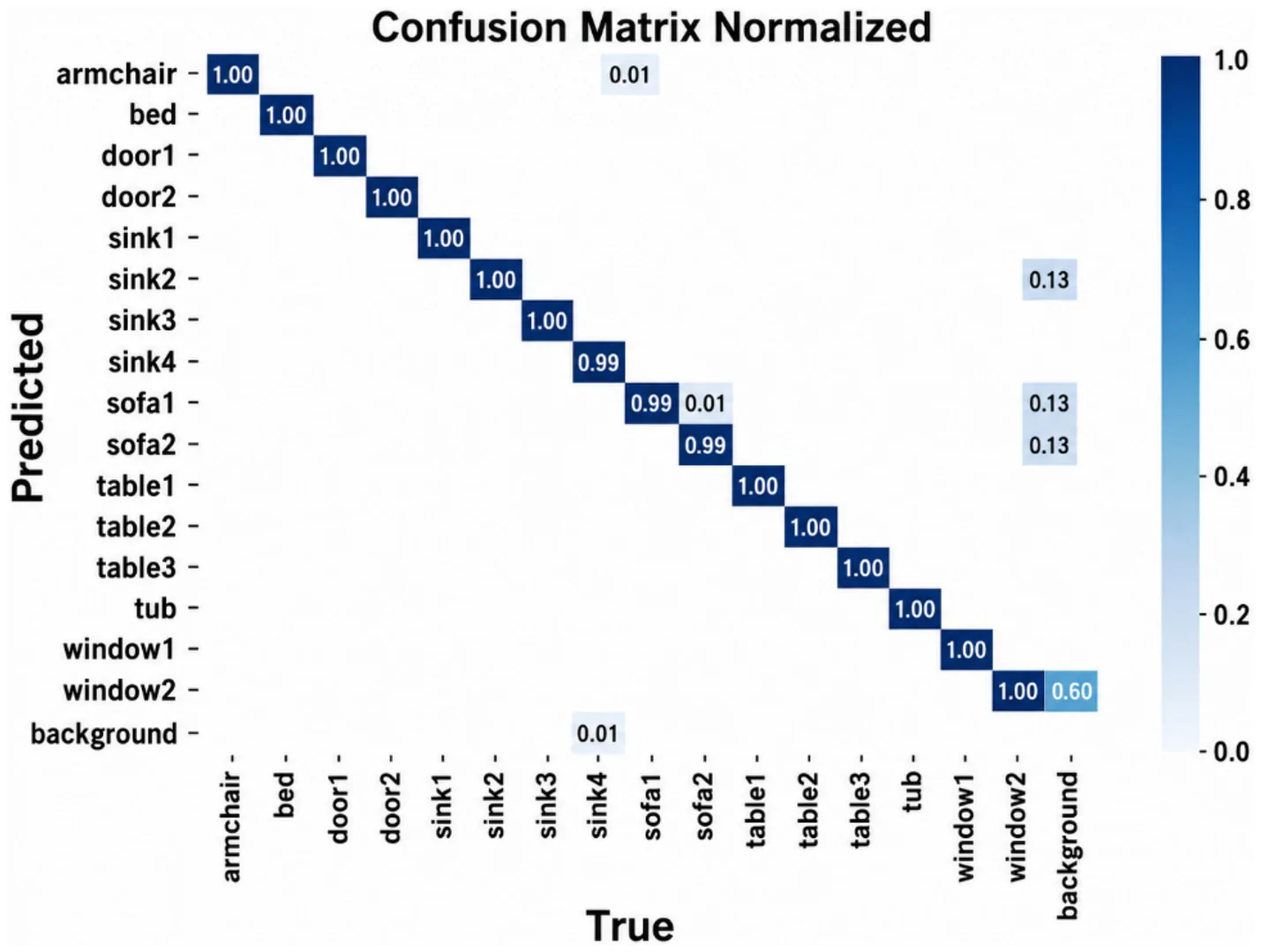
SESYD confusion matrix.

**Figure 6:**
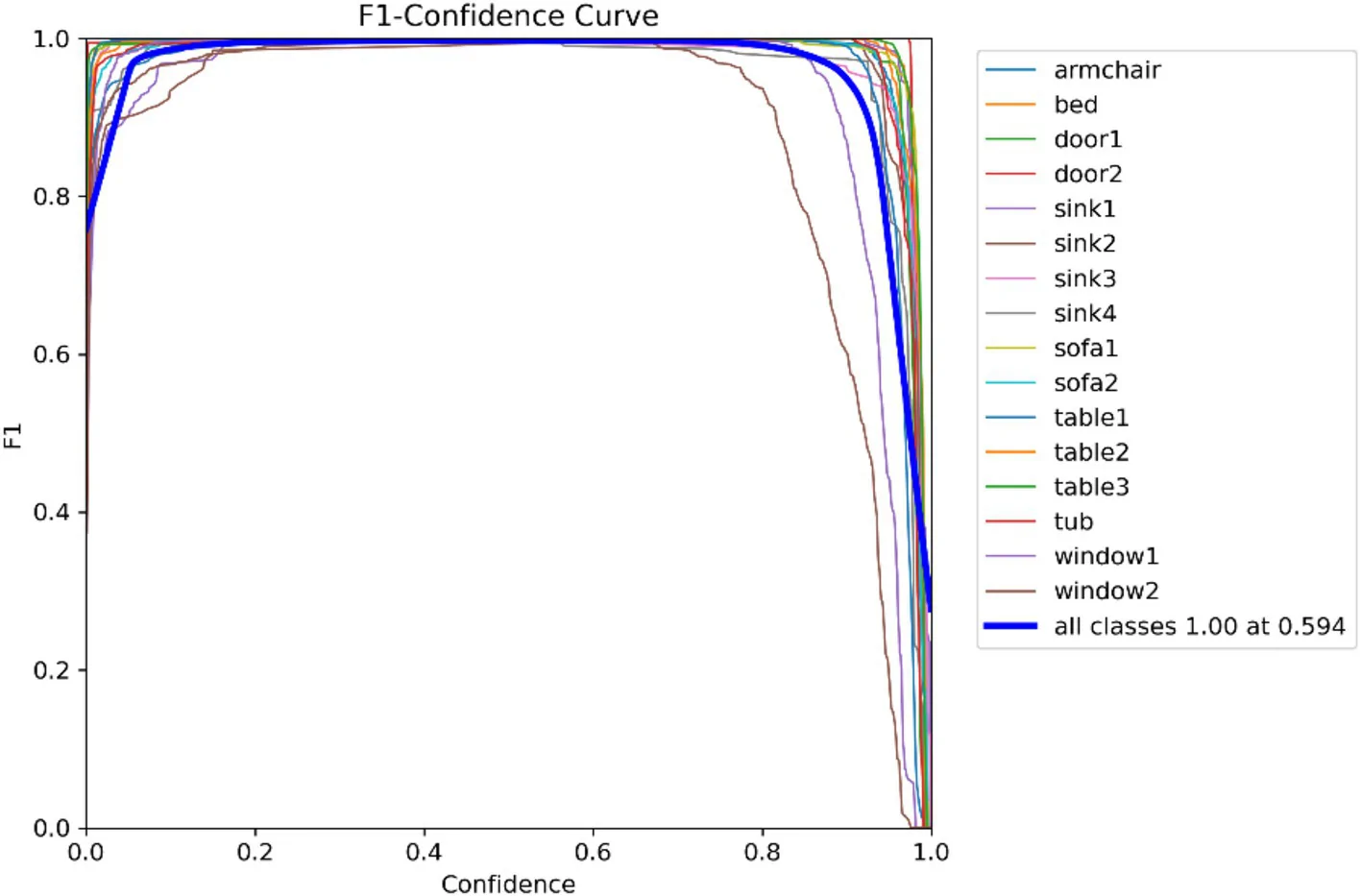
F1 confidence curve.

**Figure 7:**
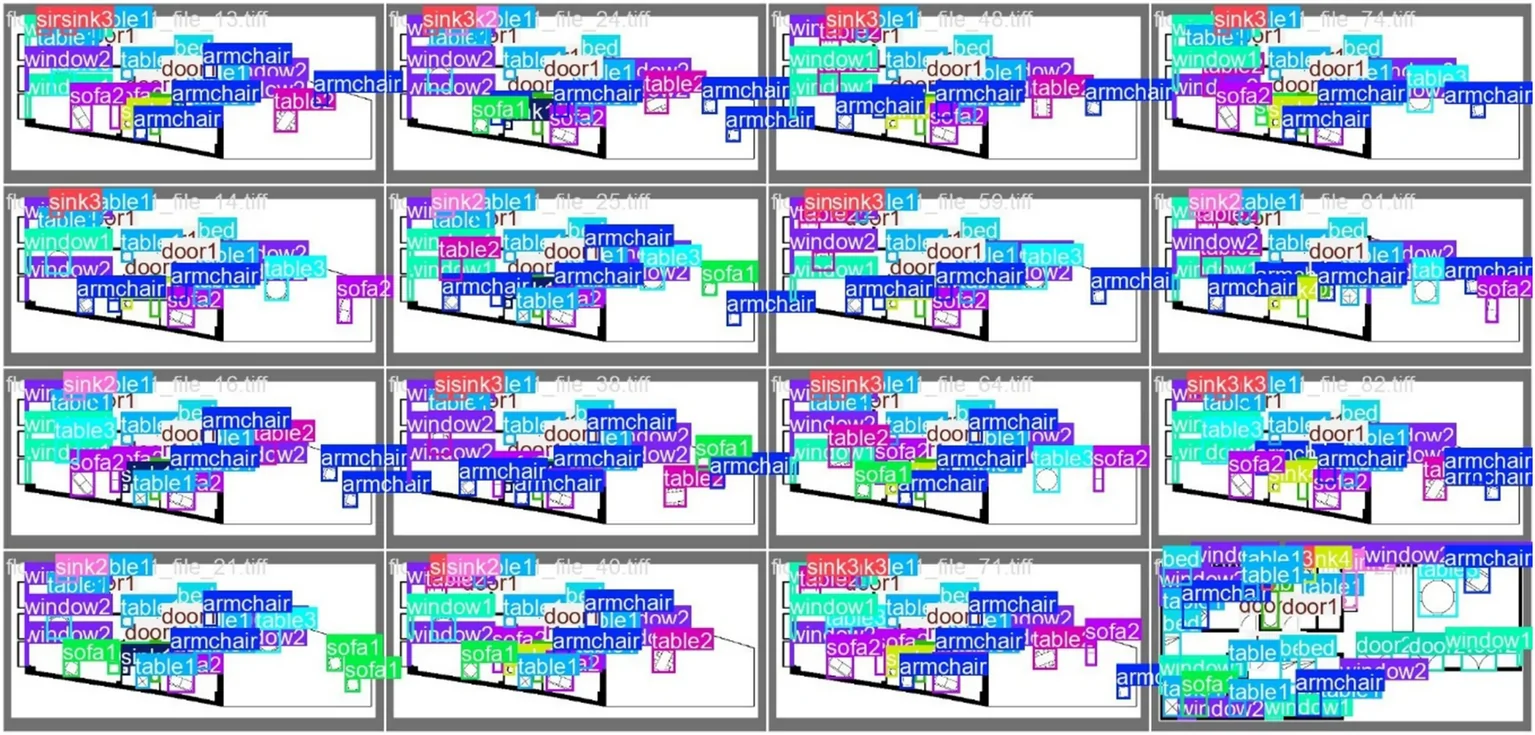
Detection results.

#### 4.3.3 Ablation study on SEYSD dataset

The use of CBAM results in a clear decrease in all metrics, and the mAP 0.5:0.95 drops to 0.8724, while recall drops to 0.9582. The drop is not only due to limited training data but also to a mismatch between CBAM’s spatial attention mechanism and the statistics of the floor plan image. CBAM’s spatial branch is created by channel-wise max- and average-pooling of activation maps, which assumes meaningful spatial variation in local activation intensity in the case of natural images. In floor plan activations, in contrast, the lines are thin and low in variance, set against a mostly empty background. The following 7 × 7 convolution for spatial mask creation is of a size larger than the typical line width and tends to smooth or blur this sparse boundary signal, rather than sharpen it. As a result, CBAM’s spatial attention suppresses the thin line cues the model is using to distinguish the symbols, as seen in the decrease in recall (0.9582) and mAP@0.5:0.95 (0.8724) in Table 2.

This study shows that it is not what one adds but rather where one adds it that is more important. In principle, CBAM’s dual attention pathway is more expressive, but it seems it hampers feature learning in the sense that it seems to wash it away rather than accentuate it, especially for floor plan images that are already structurally regular because they contain a lot of symbols. The application of ECA in both C2f and SPPF shows better performance than the baseline, although the improvement is not significant enough to address the need for re-calibration of the channels at the pooling stage as it is mostly redundant in the backbone. Using C2f blocks, the lightweight efficiency of YOLOv8n is retained, and the precision, recall and mAP@0.5:0.95 gains are most consistent, reinforcing the notion that selective attention is better than all-encompassing attention.

We further compare vanilla ECA embedded at three places [backbone-only, neck-only, and combined (C2f + SPPF)] with FloorYOLO. Backbone-only placement is the best performing vanilla placement across mAP@0.5:0.95 (0.976), beating both backbone-and-neck (0.9754) and neck-only placement (0.972). This means that recalibration benefits the most in the backbone where symbol-level features are initially generated and extend backward into the PAN-FPN neck do not provide further benefit and slightly degrade performance compared to using backbone only. Comparing this backbone-only vanilla model with the FloorYOLO model, we observe an additional performance gain (mAP@0.5:0.95 of 0.9794 vs. 0.976; precision of 0.9984 vs. 0.996), indicating that the residual connection brings additional and smaller gains on top of the placement effect. The results of these comparisons suggest that the backbone layers are the key to FloorYOLO’ s performance and the residual connection layers are just an additional refinement.

To evaluate whether FloorYOLO’ s backbone-based attention design improves robustness to challenging failure modes in floor plan images, rather than merely yielding marginal improvements in overall performance, we selected three challenging subsets from the SESYD test set that have been measured according to the size and contrast statistics of the objects: ultra-small symbols with less than a 65.7px bounding-box threshold (*n* = 145), densely overlapping instances (*n* = 134), and low-contrast line structures below a contrast threshold of 61.5 (*n* = 30). The mAP differs by only 0.35 points on the small-symbol subset (from 0.9742 to 0.9777) and has an associated precision increase, suggesting that channel recalibration enables the channel to capture discriminative features for symbols with restricted spatial extent. For the “overlap subset” the FloorYOLO achieves an mAP@0.5:0.95 of 0.9810, which is 0.33 points higher than 0.9777 of the unmodified models; in addition, it achieves a perfect recall of 1.0 for the “overlap subset,” indicating that the attention mechanism helps to disambiguate spatially adjacent instances. On the low-contrast subset, both models already exceed 0.986 mAP@0.5:0.95, and FloorYOLO shows a marginal recall trade-off (1.0000 → 0.9984) alongside a negligible change in mAP@0.5:0.95 (0.9867 → 0.9866). It means that, there are much less contrast structures at the baseline performance for this dataset and that this is not the main contrast bottleneck solved by channel attention; this subset is also the smallest (*n* = 30) which limits the statistical power of this comparison. Overall, the hard-sample results reveal that the enhancements of FloorYOLO are focused on the failure modes of small objects and overlaps that are relevant to the floor-plan symbol detection problem itself, rather than a uniform shift across the entire dataset.

To confirm that all models converged to a stable optimum within the set number of epochs (30 epochs), we present full training and validation loss curves (box, classification, and distribution focal loss) alongside precision/recall as well as the mAP@0.5 and mAP@0.5:0.95 per epoch for all models discussed in this ablation, including the FloorYOLO (Figure 8), vanilla ECA (Figure 9), backbone-only (Figure 10) and neck-only attention placements (Figure 11), and CBAM (Figure 12). The mAP@0.5:0.95 values of all variants tend to level off and the step change around epoch 20 is not caused by training instability and is a result of the switch from using mosaic augmentation to no mosaic augmentation (close mosaic = 10). The CBAM variant results in lower final accuracy as reported in Table 5 and has significantly higher loss at epoch 30 than all other variants, while precision/recall shows a more erratic trend during training, which is consistent with this variant experiencing more difficulty in optimizing during this epoch budget compared to the other variants with lower losses and more stable results (see Figure 13).

**Figure 8:**
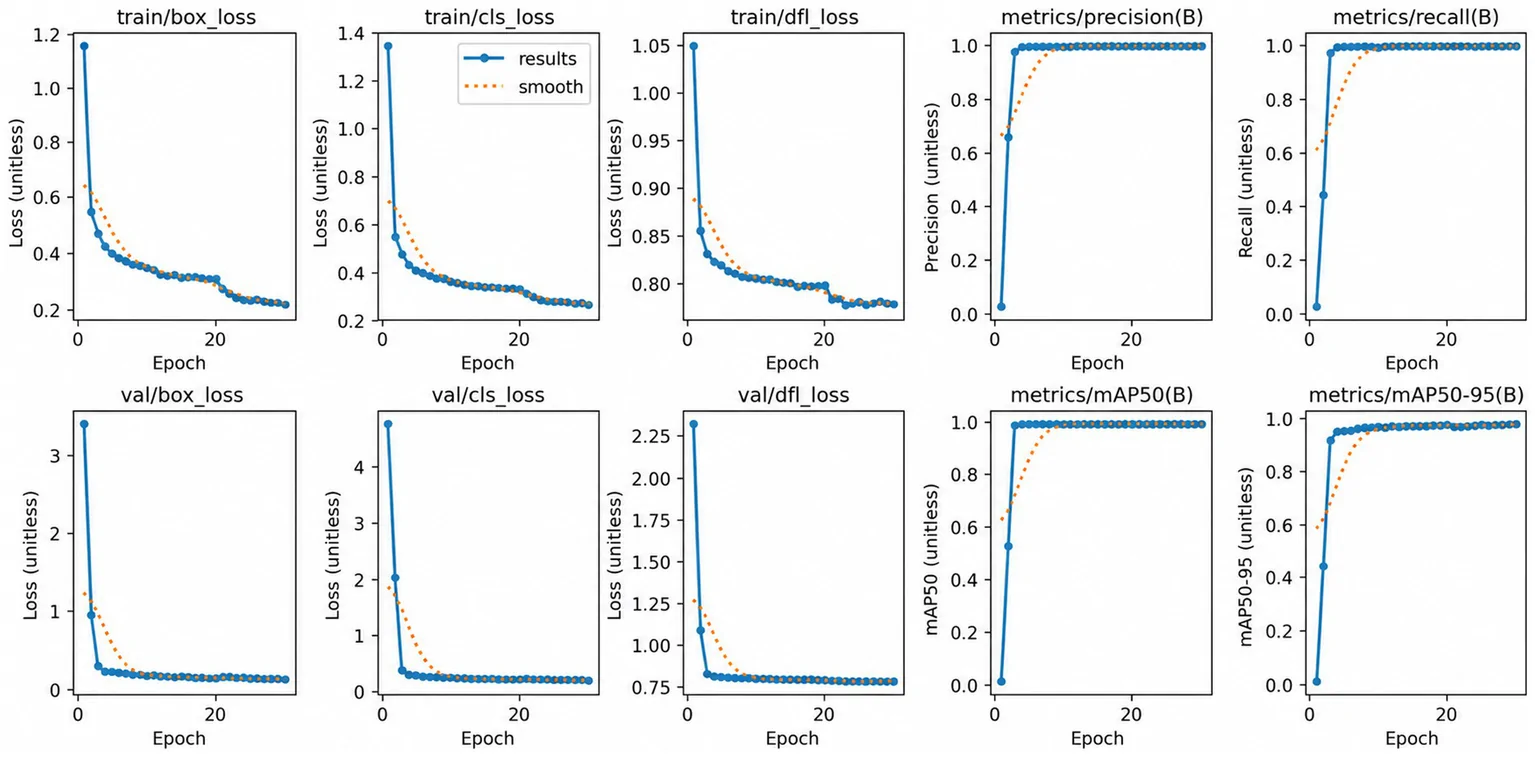
FloorYOLO (ECA).

**Figure 9:**
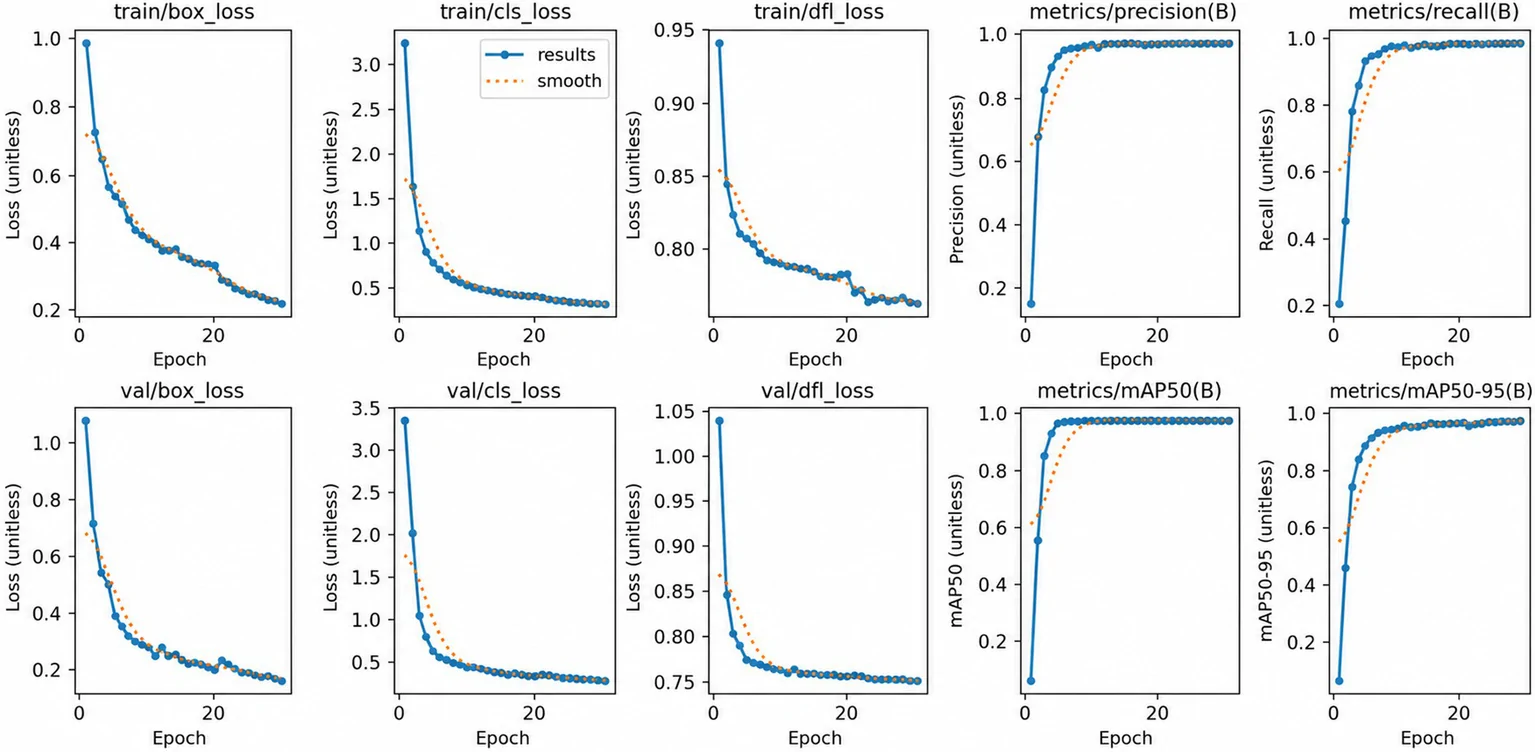
Vanilla ECA.

**Figure 10:**
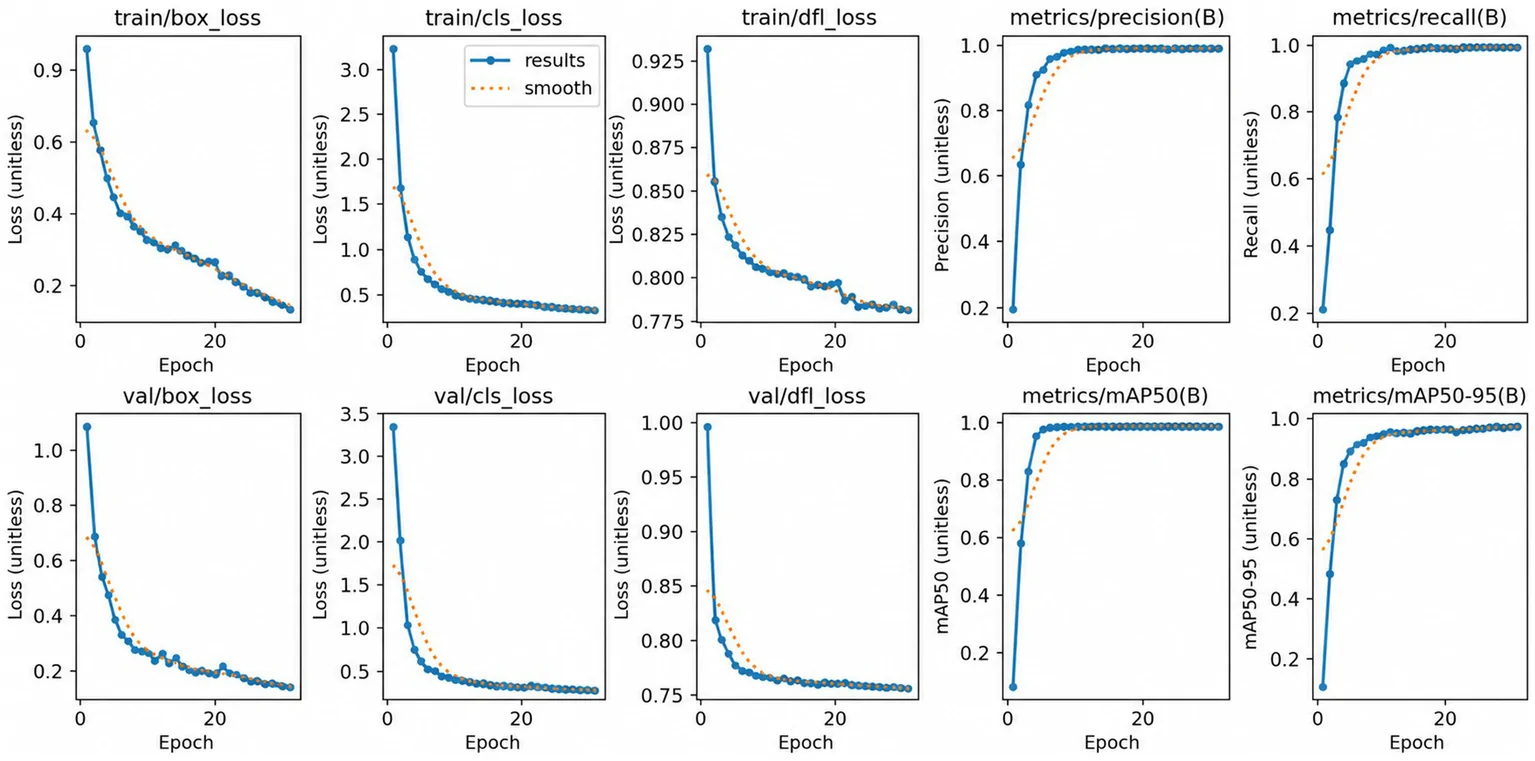
ECA in backbone.

**Figure 11:**
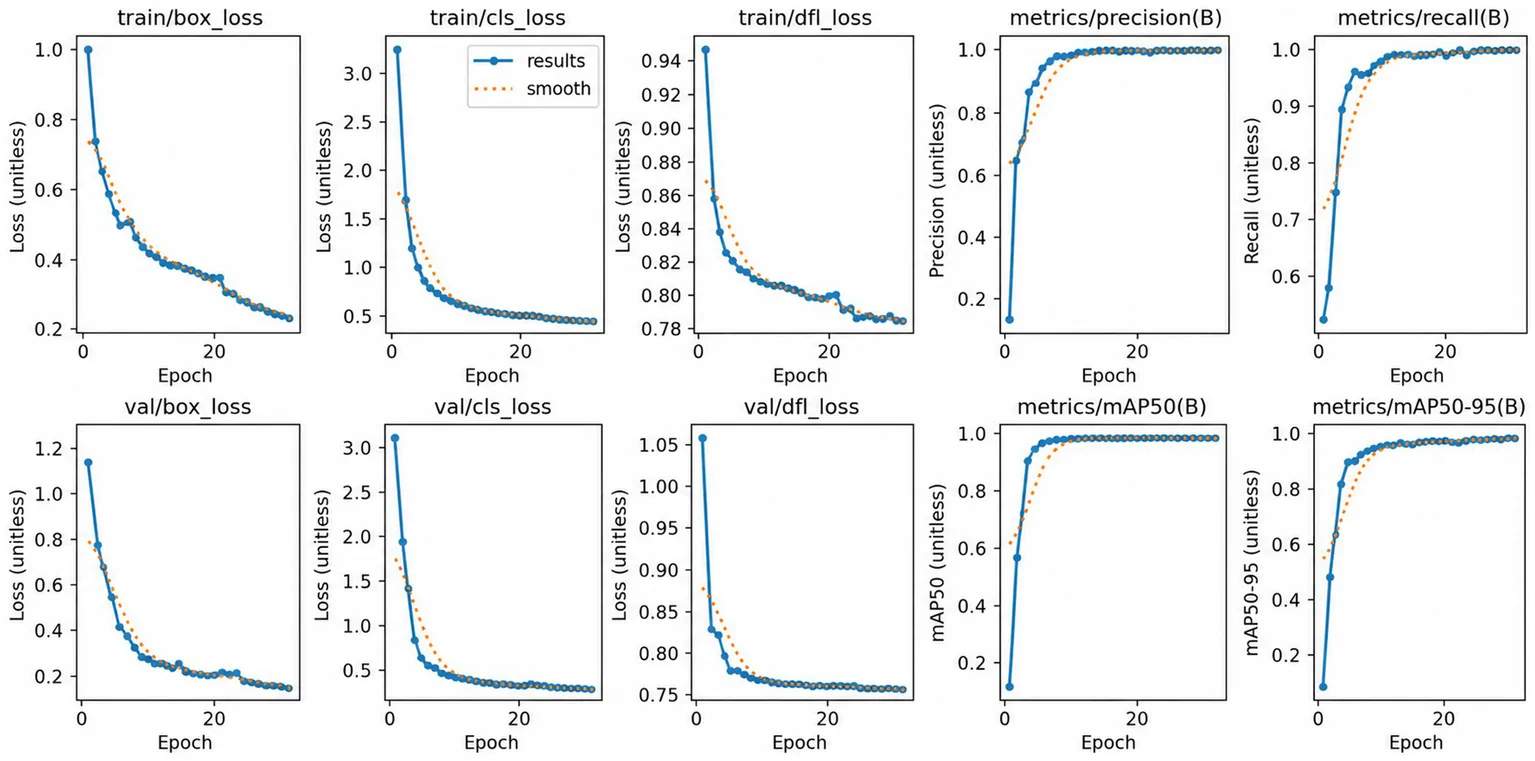
ECA in neck.

**Figure 12:**
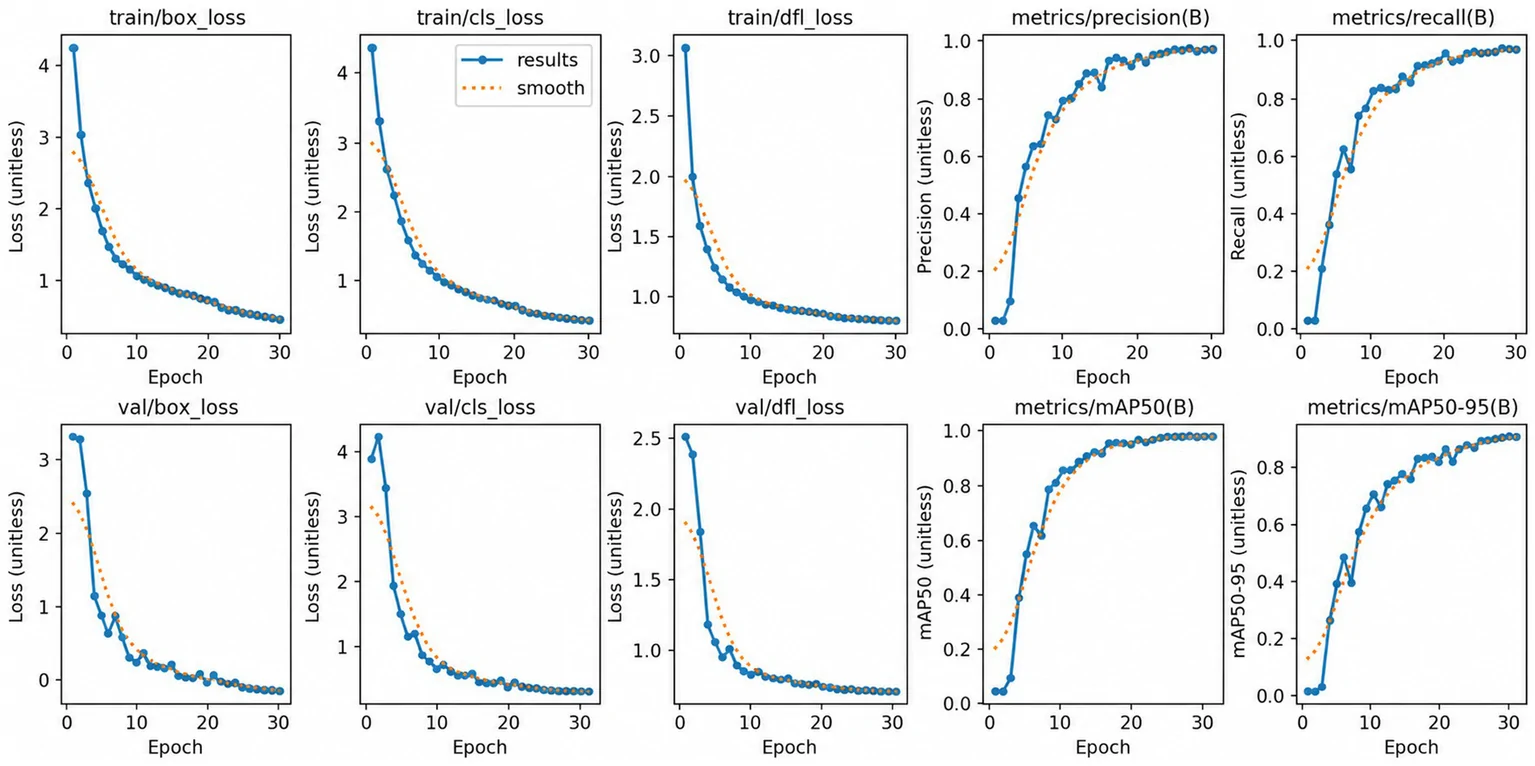
CBAM.

**Figure 13:**
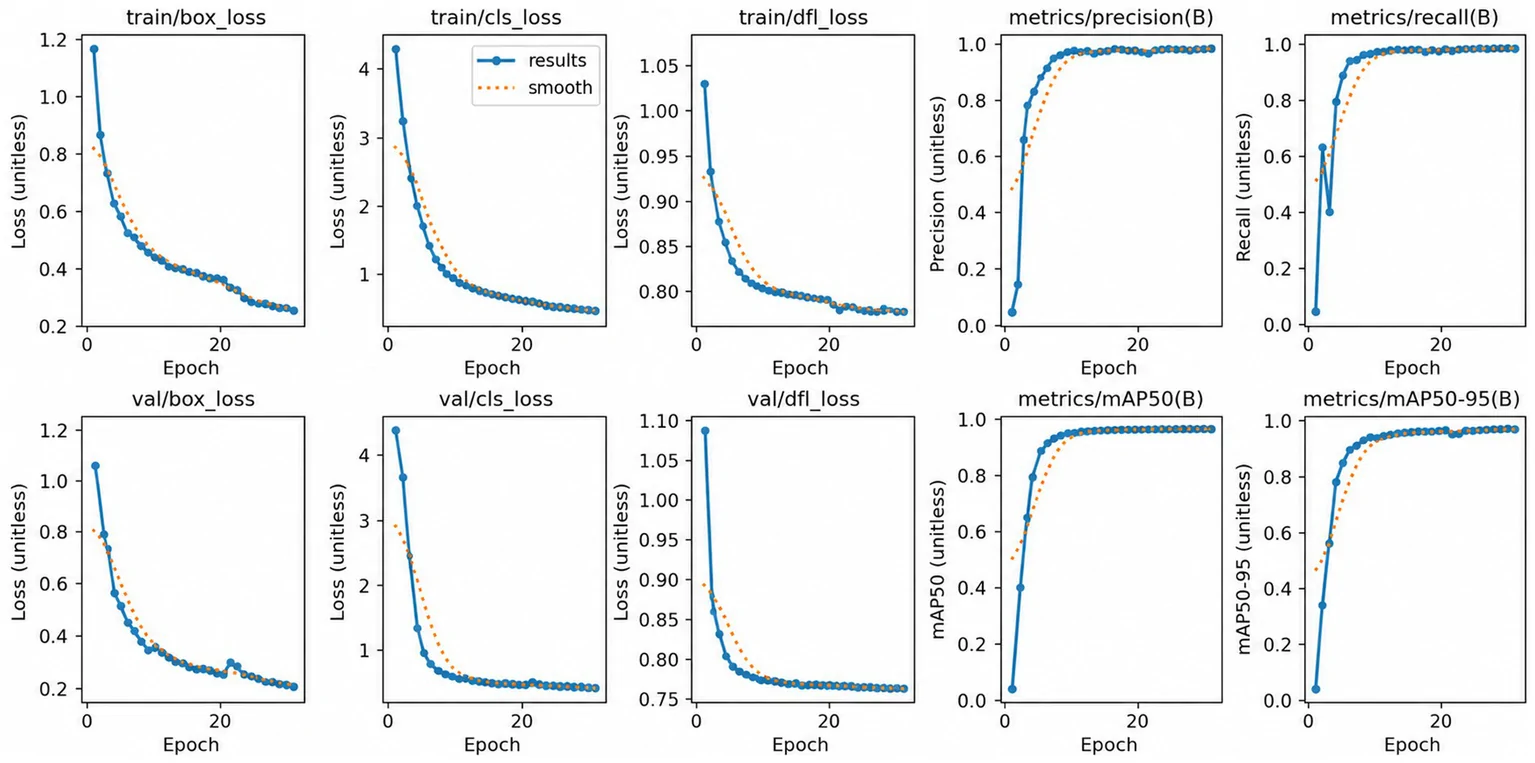
ECA on all c2f + SPPF.

#### 4.3.4 Evaluation of other models

Table 3 shows the proposed framework and existing object detection methods used for the floor plan dataset of SESYD. Ziran and Marinai (2018) used Faster R-CNN variants on the d1/d2 subsets of SESYD and reported a comparatively low mAP@0.5 of 0.31–0.39, which is due to the challenge of small, densely arranged architectural symbols. To boost performance further, Rezvanifar et al. (2020) used a tiling-based inference strategy and a YOLOv2 backbone with the help of a ResNet-50 network, obtaining an mAP@0.5 of ~0.981. Mishra et al. (2021) also used a Cascade Mask R-CNN trained on a merged SESYD and SFPI corpus to get the mAP@0.5 of 0.982. To achieve the best result that had been reported so far, Shehzadi et al. (2022) used a semi-supervised Mask R-CNN with a student-teacher model, which achieved the mAP@0.5 of about 0.995. Compared to these heavier two-stage and semi-supervised pipelines, the proposed FloorYOLO model with a single-stage and light architecture achieves an mAP50 of 0.9950, in comparison to these complex pipelines, which can achieve the same mAP50 with much fewer parameters and lower inference costs.

#### 4.3.5 Computational efficiency analysis

To assess the model’s lightweight design and suitability for real-time edge deployment, we report its number of parameters, GFLOPs, and inference speed (GPU and CPU) along with the accuracy of detection in Table 6. The models in this comparison were trained for 30 epochs to have a fair comparison. Compared to baseline: FloorYOLO has the same number of parameters (3.014 M) and GFLOPs (8.208) as YOLOv8n and achieves an improved mAP@0.5:0.95 of 0.9794 compared to 0.9740. GPU throughput drops by only ~1.2% (97.79 → 96.66 FPS) less than the baseline, and CPU throughput is only ~5.2% (6.60 → 6.26 FPS) less than the baseline, confirming the accuracy gain comes at effectively zero additional computational cost.

Compared to a classic lightweight CNN detector, MobileNet-SSD has the smallest number of measured parameters (2.414 M) and the highest CPU throughput (13.92 ± 1.28 FPS) about 2.2X faster than FloorYOLO on CPU. But the mAP@0.5:0.95 of 0.7723 is over 20 points lower than FloorYOLO’ s 0.9794 and the GPU throughput (53.63 FPS) is lower than FloorYOLO’ s (96.66 FPS) even though the number of parameters is smaller and so indicating fewer parameters alone do not guarantee faster or more practical inference (see Table 7).

**Table 7:** Ablation study results on CVC-FP dataset.

Model	Split	mAP@0.5	mAP@0.5:0.95	Precision	Recall
Baseline (YOLOv8n)	Val	0.8116	0.4695	0.808	0.7405
FloorYOLO	Val	0.8241	0.4805	0.8146	0.7570

To compare with one of the smallest publicly available detectors, NanoDet-m (320 × 320 input, 0.95 M parameters, 0.72 GFLOPs) is also tested with the same 30 epochs training schedule as all the other detectors compared. The mAP@0.5:0.95 is as low as 0.451, which is significantly lower than FloorYOLO’ s 0.9794. The gap is not uniform, per-class analysis reveals that door classes achieve 64.8–67.5% mAP, whereas thin structural elements, like windows, achieve 7.8–8.7% mAP. This implies NanoDet-m’s very small backbone, which works well across generic object classes, but fails to perform well on fine, thin, and visually similar structural symbols that are typically found in floor-plan drawings, which is exactly the problem FloorYOLO’ s design aims to address.

The results, together with the RT-DETR-L comparison in Section 4.3.4, show that the FloorYOLO favors the accuracy-efficiency trade-off compared to both classic lightweight CNN detectors and larger transformer-based detectors, proving the “lightweight” and “real time edge deployment” described in this paper.

### 4.4 Performance evaluation of FloorYOLO model on the CVC-FP (computer vision center floor plan) dataset

#### 4.4.1 CVC-FP dataset

CVC-FP (Computer Vision Center Floor Plan) dataset is made up of synthetic floor plans reflecting the real architectural environment. The elements are encoded with polygons in SVG format. This dataset contains various images of floor plans having different objects such as rooms, doors, windows, and tables. There is a total of 123 images available in this dataset with complex architectural designs. These floor plans are not labeled and have been used in Floor Plan Recognition studies. The dataset includes two furniture classes, one door class, and one window class distributed across various rooms (see Figures 14, 15).

**Figure 14:**
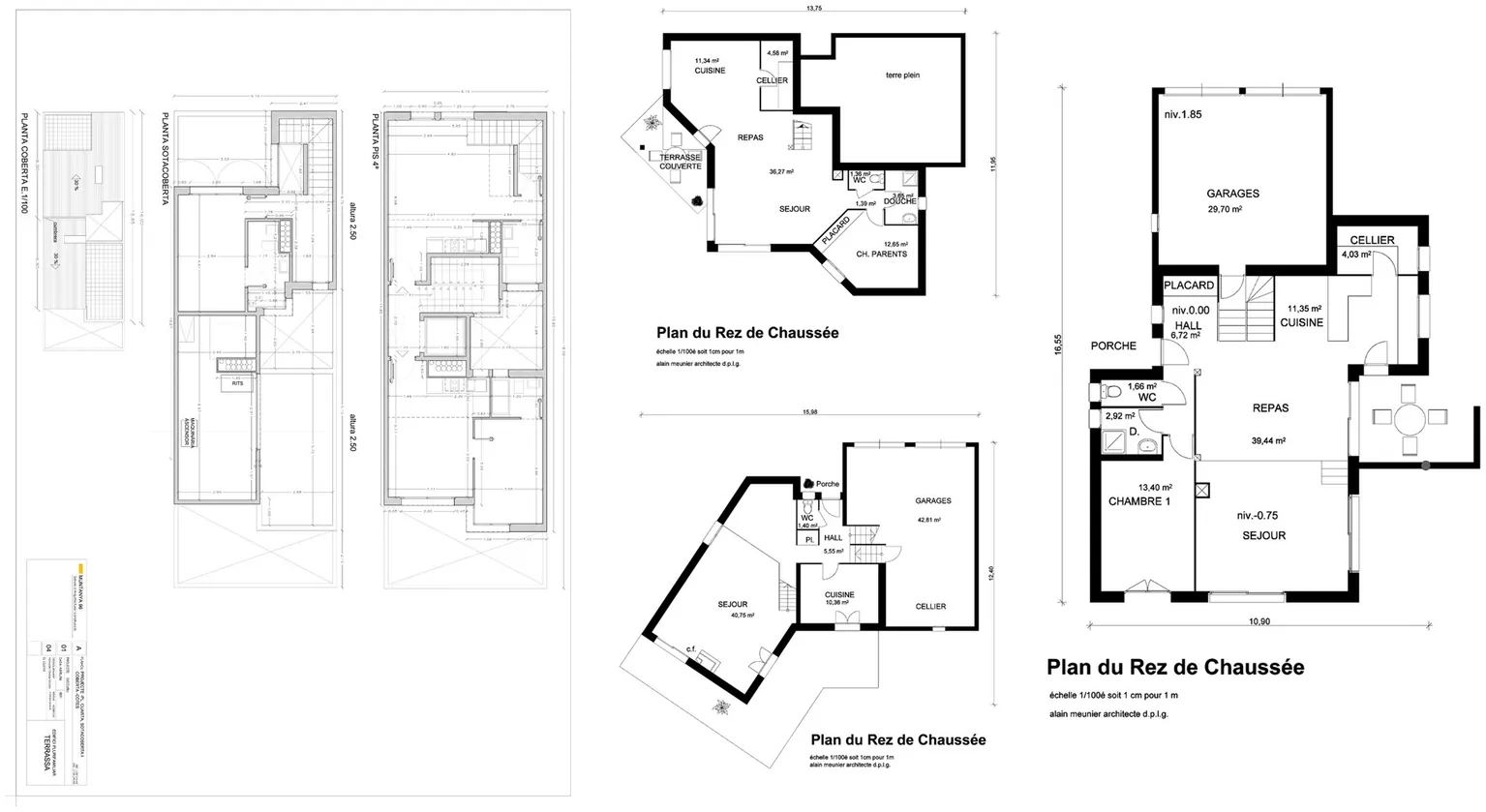
Sample CVC-FP floor plan images.

**Figure 15:**
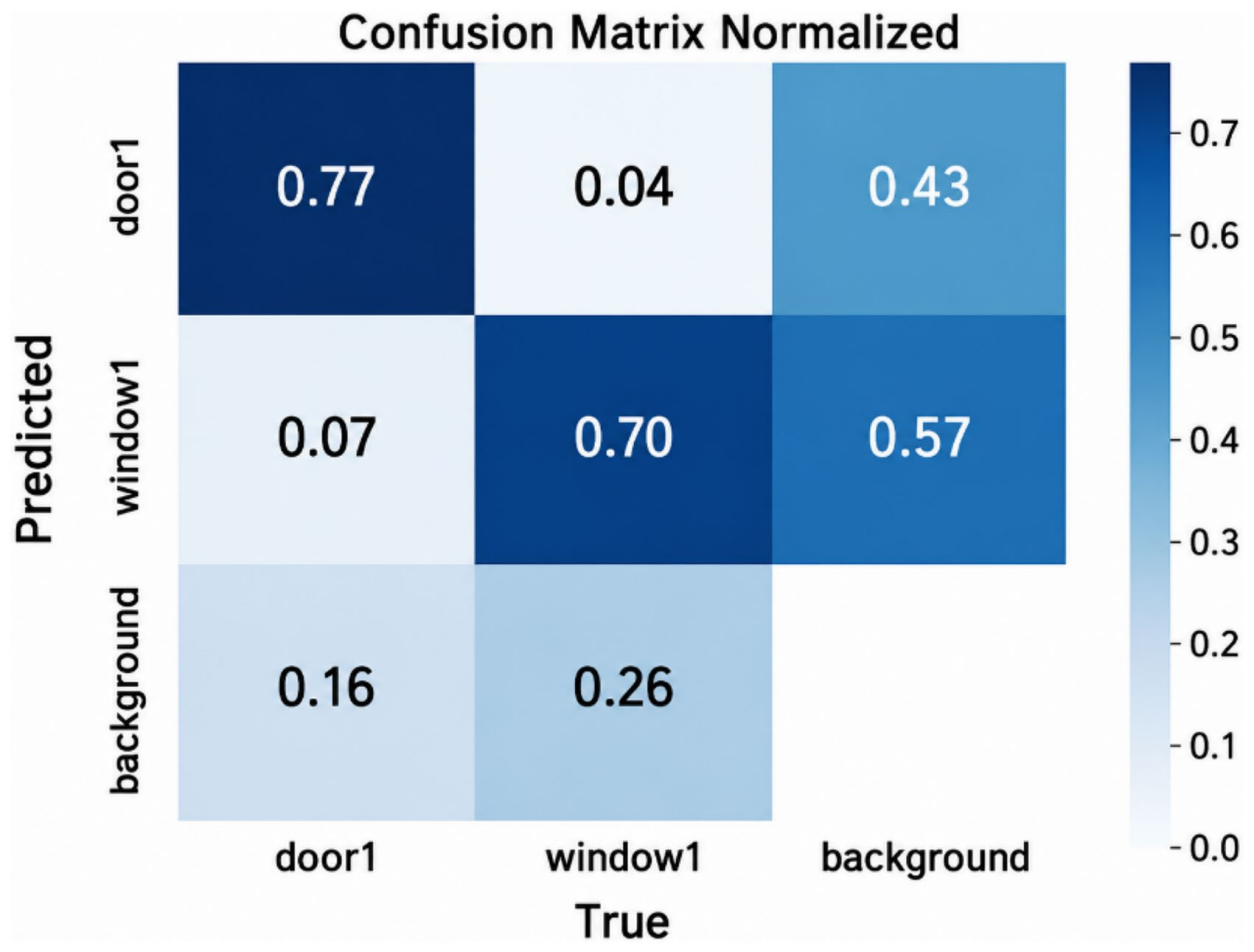
CVC-FP confusion matrix.

#### 4.4.2 Performance evaluation of proposed network on CVC-FP

We evaluate YOLOv8n with an ECA-based network on a floor plan dataset using 98 images for training and 25 images for validation. Figure 16 shows the average precision of every class separately. The per-class validation results show that door1 achieved a Box Precision of 0.810, Recall of 0.786, and mAP@0.5 of 0.871, while window1 achieved a Box Precision of 0.791, Recall of 0.727, and mAP@0.5 of 0.777. The overall validation performance of the FloorYOLO model achieved an mAP@0.5 of 0.8241 and mAP@0.5:0.95 of 0.4805, with a Precision of 0.8146 and Recall of 0.7570. Compared to the YOLOv8n baseline, which recorded an mAP@0.5 of 0.8116, mAP@0.5:0.95 of 0.4695, with Precision of 0.808, and Recall of 0.7405, the FloorYOLO model demonstrates consistent improvements across all key metrics, confirming the benefit of channel attention for architectural element detection in floor plan imagery (see Figure 17).

**Figure 16:**
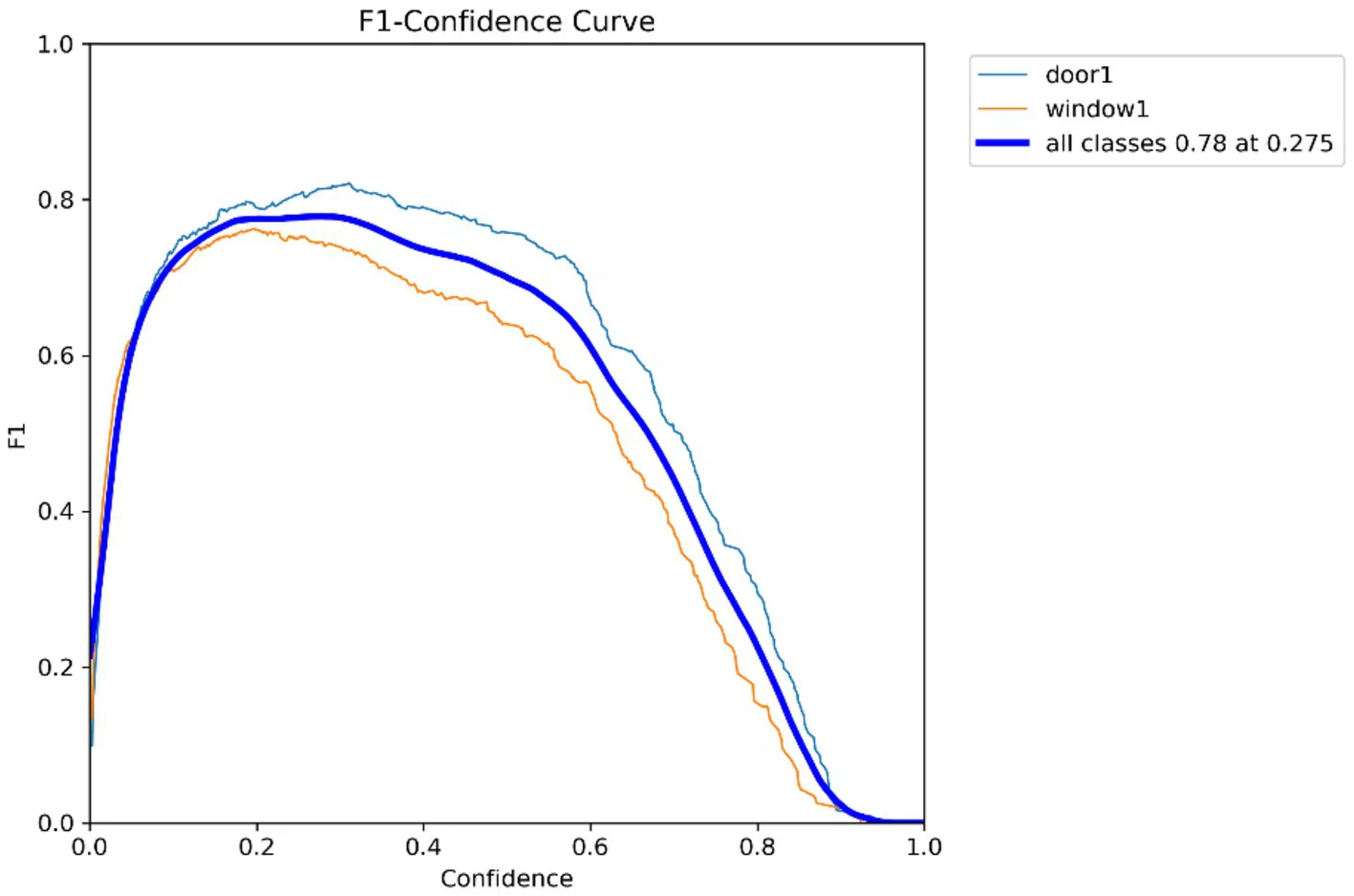
CVC-FP F1 curve.

**Figure 17:**
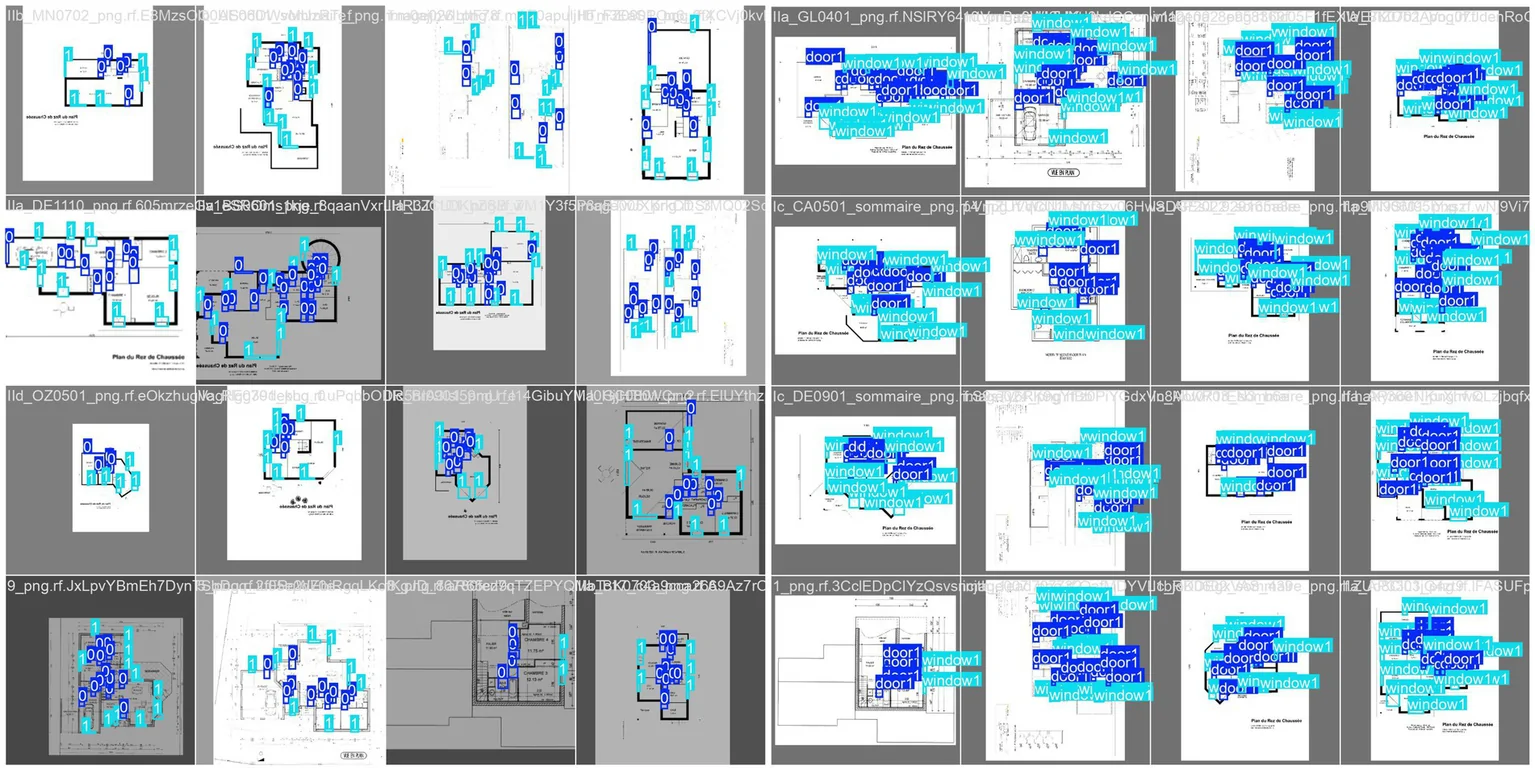
CVC-FP detection results.

#### 4.4.3 Ablation study on CVC-FP dataset

The ablation study on the validation set shows that the overall detection performance is enhanced by adding the Efficient Channel Attention (ECA) mechanism into all the C2f layers. The ECA enhanced model gives higher mAP@0.5 and mAP@0.5:0.95 values which are 0.8241 and 0.4805, respectively. Furthermore, the precision and the recall are raised to 0.8146 and 0.7570, respectively, to get better feature discrimination and stable detection performance. The results support both localization accuracy and prediction consistency with channel attention incorporation.

#### 4.4.4 Evaluation of other models

Table 8 shows the difference between the proposed model and the previous models that are evaluated using the CVC-FP dataset. Dodge et al. (2017) proposed a pipeline that utilizes fully convolutional networks for wall segmentation, Faster R-CNN for architectural object detection and OCR for text extraction, with a mean IoU of 94.4 for the wall-parsing stage on CVC-FP. Due to the structural differences between the floor plans of CVC-FP and SESYD, the lower image resolution and the varying drafting conventions, Ziran and Marinai (2018) directly evaluated Faster R-CNN and SSD on CVC-FP with a VGG-16 network achieving a mAP score of 0.31 on the d1 subset and 0.39 on the d2 subset. The results emphasize the challenges that the conventional CNN-based detectors have in adapting to different floor plan styles without dataset-specific fine-tuning or additional segmentation or OCR steps. Under the same CVC-FP setting, the proposed FloorYOLO model shows that an attention-augmented single stage detector could outperform the multi-module pipeline detector and the heavyweight backbone like VGG-16.

**Table 8:** Comparison with previous CVC-FP floor plan detection methods.

Title	Author (year)	Model	Dataset	Metrices
Parsing floor plan images	Dodge et al. (2017)	FCN (wall seg.) + Faster R-CNN + OCR	CVC-FP	Mean IoU = 94.4
Object detection in floor plan images	Ziran and Marinai (2018)	Faster R-CNN, SSD (VGG-16 backbone)	CVC-FP	mAP 0.31 (d1)/0.39 (d2)

#### 4.4.5 Failure analysis on CVC-FP

Figure 18 displays some failure examples from the CVC-FP test set of the YOLOv8n and FloorYOLO (ResECA) models on four floor plans with different drawing conventions. Most often, the baseline is wrong with the classification instead of the localization (YOLOv8n gets only 0% recall for the window class in Table 9, 176/234 localization instances are wrongly classified, and 58 localization instances are not detected at all). Against a baseline 168/234 (71.8%) correct identification of window instances, FloorYOLO does a substantial improvement and is able to correctly distinguish between window and door symbols in the same openings. However, at a more conservative inference confidence (conf = 0.5), the baseline yields duplicate and overlapping detections in the vicinity of door openings, even with the thin vectorized strokes of CVC-FP, which shows that the strong activations in the vicinity of the door openings are not completely suppressed by inference. Interestingly, this improvement is not achieved for all CVC-FP drawing conventions: FloorYOLO’ s recall on plans with a more technical architectural notation, such as dimension annotations and hatched walls (Figure 19), is significantly lower compared to the plans with the most common line-drawing style from the dataset, suggesting that although an attention-based recalibration works well for the dominant CVC-FP convention, it does not fully overcome the representational gap for less-represented drawing styles (see Figure 20, Table 10).

**Figure 18:**
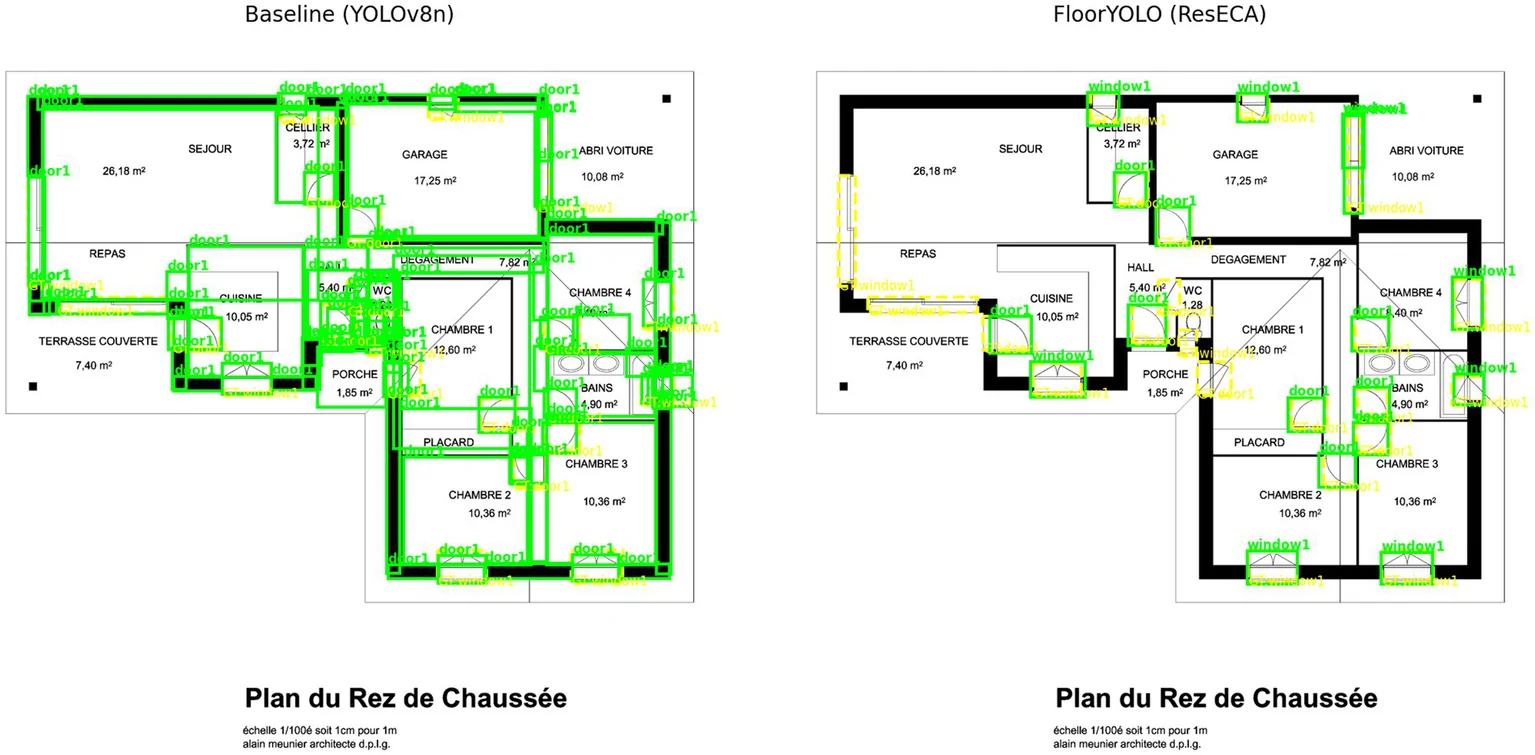
Comparison of detection results from the baseline YOLOv8n and FloorYOLO (ResECA) on a CVC-FP floor plan.

**Table 9:** Per-class error breakdown and size-stratified recall on CVC-FP (a) error type breakdown.

Model	Class	Correct	Localization	Classification	Missed	False positive
Baseline (YOLOv8n)	door1	196	31	0	17	1,540
Baseline (YOLOv8n)	window1	0	0	176	58	0
FloorYOLO (ResECA)	door1	177	3	17	47	22
FloorYOLO (ResECA)	window1	168	6	7	53	27

**Figure 19:**
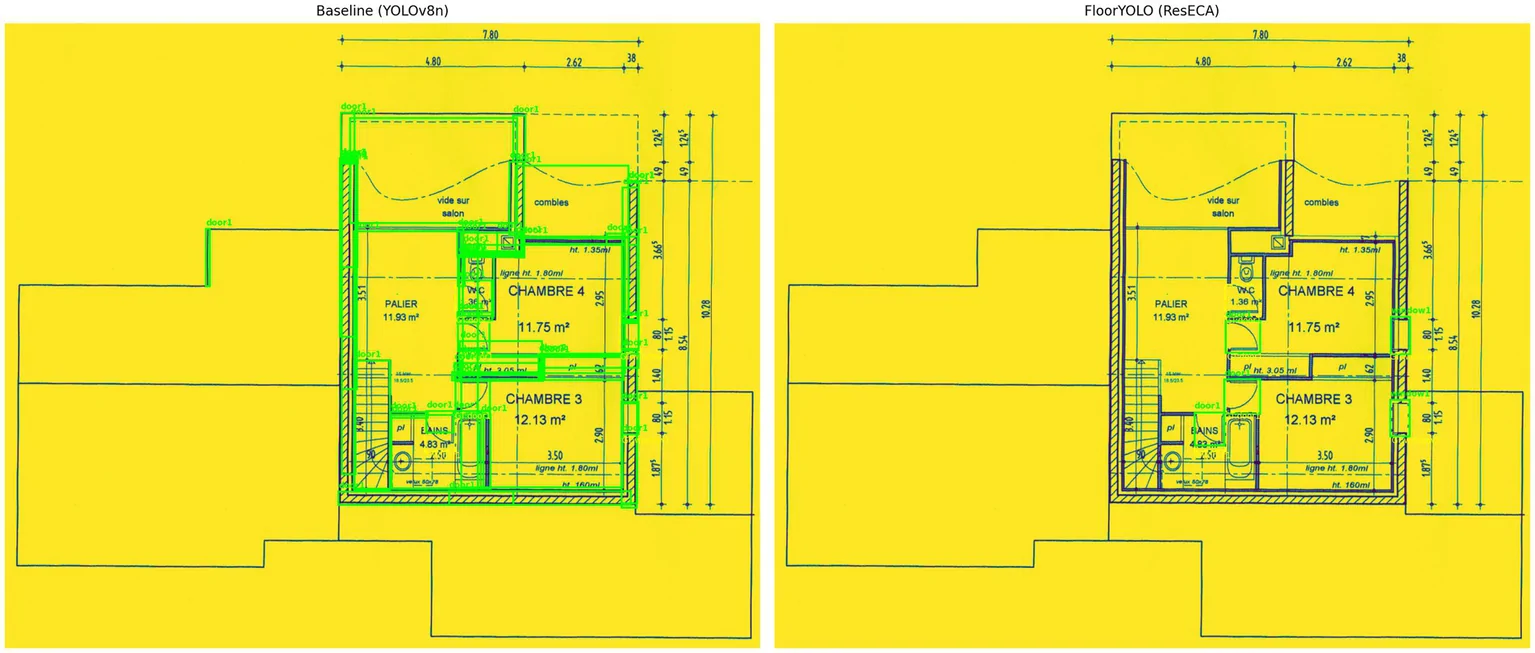
Comparison of detection results from the baseline YOLOv8n and FloorYOLO (ResECA) on a CVC-FP floor plan with technical architectural notation.

**Figure 20:**
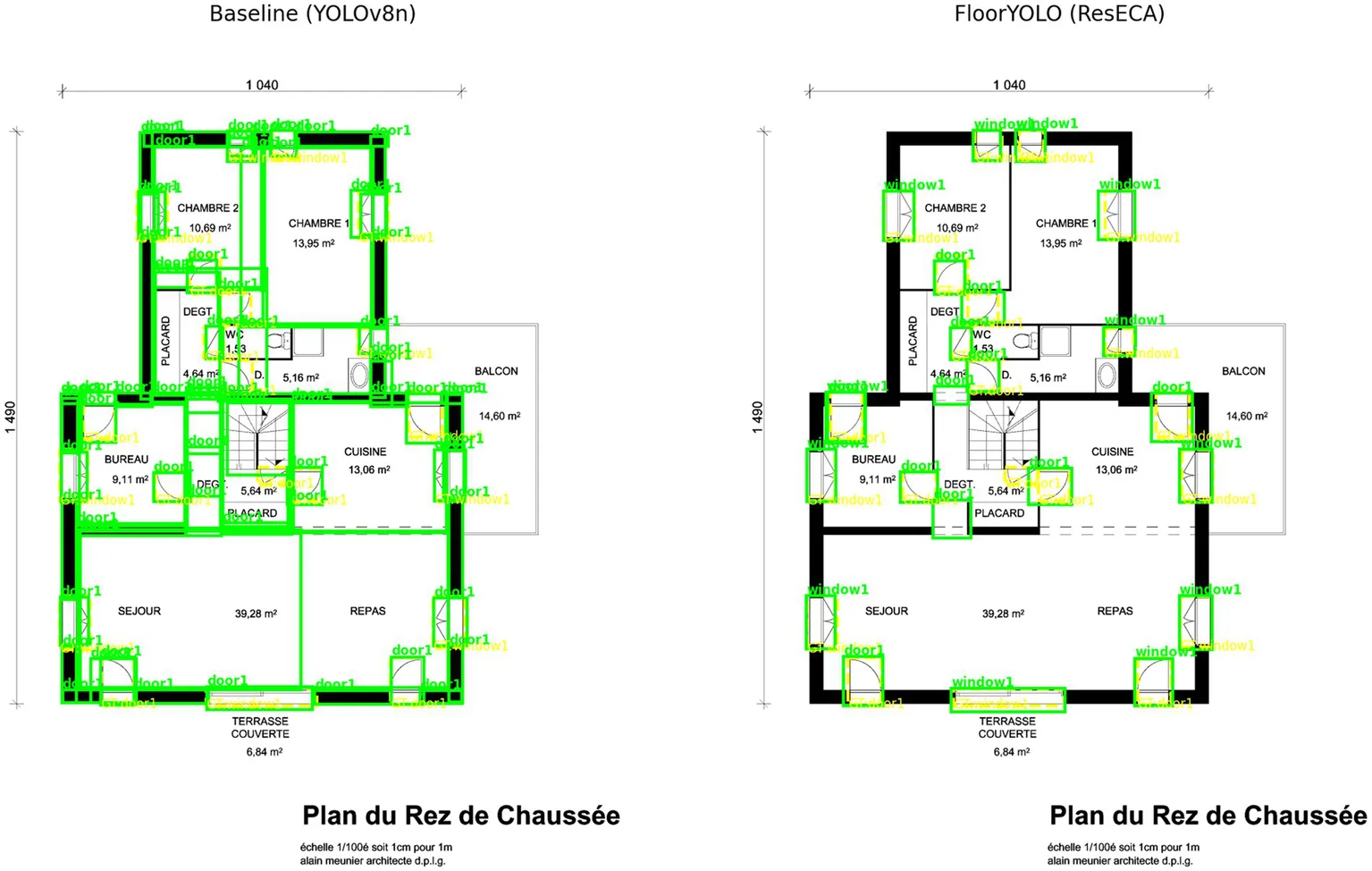
Comparison of detection results from the baseline YOLOv8n and FloorYOLO (ResECA) on a CVC-FP floor plan with a common line-drawing style.

**Table 10:** Per-class error breakdown and size-stratified recall on CVC-FP (b) recall by object size.

Model	Small	Medium	Large
Baseline (YOLOv8n)	—	0.208	0.486
FloorYOLO (ResECA)	—	0.715	0.724

## 5 Discussion and future work

The proposed method is based on a YOLOv8n algorithm with the addition of the Efficient Channel Attention (ECA) mechanism designed for the detection of structural components in the architectural floor plans: rooms, doors, windows and parts of furniture. The model combines low-weight channel attention into all C2f layers to improve the feature representation and discriminative ability of the model, overcoming the limitations of the existing floor plan recognition method in complex and irregular floor plan recognition. The experimental results on the SESYD and CVC-FP datasets show that the model outperforms the baseline model in terms of mAP, precision, and recall. The structural and furniture element information obtained by this method has the potential to aid multiple downstream processes such as automated generation of Building Information Modelling (BIM), room type labelling and segmentation, Augmented Reality (AR) based indoor navigation, evacuation and emergency egress planning, and 3D floor plan reconstruction, which are discussed in further detail below. While these enhancements have been made, the extra attention mechanism adds extra computation load, potentially restricting its use in resource-constrained settings. Additionally, the datasets employed to evaluate (SESYD and CVC-FP) are not representative across building scales and functional types (e.g., commercial or institutional) and the reported results should be interpreted within the scope of the evaluated datasets.

Beyond dataset size, there is also a domain shift between SESYD’s vector-rendered synthetic floor plans and CVC-FP’s scanned real-world floor plans, as seen in Section 4.4, where the mAP@0.5:0.95 drops from 0.9794 to 0.4805. A possible future direction is to investigate domain randomization (DR) by adding noise (such as scan-like noise), contrast levels and line widths to the SESYD training images to better simulate the variations of CVC-FP training images. Likewise, a style transfer pre-processing step between the vector-rendered style and the raster-scanned style of the floor plan could fill the visual gap prior to fine-tuning the result. One approach to address this issue is to train the model first on the SESYD dataset and then on the CVC-FP dataset. This would allow the channel-attention representations obtained from the larger SESYD dataset to better transfer to the noisier CVC-FP domain than the current study, where different models were trained on each dataset independently.

This study offers both academic and engineering value that extends beyond the reported detection metrics to the recognition outputs themselves. From the perspective of academic research, this paper provides an empirical example of the use of light-weight channel-attention mechanisms in less studied object-detection scenarios, such as monochrome image domains characterized by sparse object distributions and line-dominated structures, but similar structures to technical line drawings and engineering schematics. From an engineering perspective, the structural elements and furniture symbols detected by the proposed method can support a range of practical applications such as: speeding up the conversion of legacy scanned or manually drawn floor plans into structured digital information for semi-automated BIM (Building Information Modelling) creation, supporting space utilization analysis and initial compliance assessments for facilities management, aiding automated evacuation route planning (where locating doors and windows accurately is important), and supporting automated room and furniture recognition for real estate and interior design platforms. These applications illustrate that the method’s value extends beyond benchmark accuracy to practical deployment contexts. These downstream pipelines have not yet been implemented or empirically validated in this study; their integration and evaluation remain directions for future work.

Future research encompasses various domains of architectural development. The Floor Plan Recognition can also be effectively implemented in efficient and quick urban planning when there are a lot of Floor Plans. It is also useful for interior designing, where information related to the Floor Plans is of great significance. This invites considerations of automating the pre-process of interior designing, thus making it simpler for the designer to make necessary decisions based on individual Floor Plans and information available on that floor. Because of advances in the area of Augmented Reality (AR), Floor Plan recognition plays an important role, as it contains spatial information. Since the room types and room elements are identified in our model, this information could be utilized in creating the Augmented Reality technology to leverage the spatial arrangement in the most optimal manner possible. Lastly, this model could also be applied while making 3D models of Floor Plans. 3D models assist in giving more information and understanding of the Floor Plans as it creates a virtual experience of the Floor Plans. The information from the Floor Plans elements could be gleaned for use in the construction of the 3D models for the Floor Plans. Comparative experiments with different building types (residential, commercial, institutional, and entertainment) using different sets of samples for each type should also be conducted in the future to evaluate the universality of the proposed method.

## 6 Conclusion

The feature representation of a detection model has been improved with the addition of the Efficient Channel Attention (ECA) mechanism in this study. The main goal is to generate semi-automatic evaluations of floor plans while decreasing the amount of manual labeling required and reducing the amount of time and labor required to complete an evaluation. The FloorYOLO model was trained using the SESYD (Semantic Dataset of Architectural Plan Layouts) and CVC-FP dataset which has floor plan data containing different complex structural patterns which exist across a variety of floor plan layouts. Utilizing ECA, the detection model will have an improved ability to recognize room types, detect furniture, and identifying structures (including structures created using irregular or irregular angles). Extracting features from a mixed floor plan set remains a difficult task; however, ECA improves both accuracy and overall detection model performance compared with traditional detection models evaluated in this paper. Future work should include the use of larger and more diverse datasets and will explore improving performance on highly cluttered or complex floor layouts.

## Data Availability

The raw data supporting the conclusions of this article will be made available by the authors, without undue reservation.
